# Genome‐Wide Analysis of the *APETALA2/Ethylene‐Responsive Factor* Gene Family in *Carthamus tinctorius L*.

**DOI:** 10.1002/pld3.70032

**Published:** 2025-01-16

**Authors:** Zheng‐Wei Tan, Dan‐Dan Lu, Yong‐Liang Yu, Lei Li, Lan‐Jie Xu, Wei Dong, Chun‐Ming Li, Qing Yang, Hui‐Zhen Liang

**Affiliations:** ^1^ Provincial Key Laboratory of Conservation and Utilization of Traditional Chinese Medicine Resources, Institute of Chinese Herbal Medicines Henan Academy of Agricultural Sciences Zhengzhou China

**Keywords:** *APETALA2/ethylene‐responsive factor*, *Carthamus tinctorius L*, expression pattern, multiple sequence alignment, transcription factor

## Abstract

The *APETALA2/ethylene‐responsive factor (AP2/ERF)* superfamily represents a class of transcription factors involved in plant growth, development, and stress responses. *Carthamus tinctorius L*., also known as safflower, is an important plant whose flowers contain carthamin, an expensive aromatic pigment with various medicinal and flavoring properties. This study aimed to elucidate the roles of these transcription factors in plant growth, metabolic regulation, and environmental adaptation in safflower, providing foundational information and theoretical support for genetic improvement and stress resilience research in this crop. In this study, we identified and characterized the *AP2/ERF* family genes in safflower through a comprehensive genomic analysis. A total of 127 *AP2/ERF* genes were identified and clustered into seven groups and 14 subgroups based on phylogenetic analysis. Multiple sequence alignment revealed that the basic region and two helical structures were highly conserved in most AP2/ERF proteins. *Cis*‐acting elements in the promoters of the *AP2/ERF* genes were analyzed, and a degree of safflower specificity was observed among different safflower species. Tissue‐specific expression analysis showed that 23, 21, 15, and 9 genes were most abundantly expressed in the roots, leaves, flowers, and buds, respectively, while only eight genes were highly expressed in all tissues examined. These results indicate that the *AP2/ERF* family genes in safflower are diverse and complex, with distinct expression patterns for different genes in different safflower species. The findings provide important fundamental data for in‐depth studies of the growth, development, and stress response mechanisms in safflower.

## 1 Introduction

Safflower *(C. tinctorius L. [Ct])* is an ancient crop belonging to the *Asteraceae* family, with a long history of cultivation worldwide. As an oil‐bearing crop, safflower was officially included in the Production Yearbook Statistical Project catalogue by the Food and Agriculture Organization of the United Nations in 1973 (Ali et al. 2021; Cao et al. 2013; Chen et al. 2020). Countries along the “Belt and Road Initiative,” including Iran, India, Pakistan, and Kazakhstan, are the main planting areas of safflower (Ali et al. 2020; Gomashe et al. 2021). Safflower seeds, described as the “king of linoleic acid,” have important effects that include reducing blood lipids and preventing atherosclerosis (Kang et al. 2023; Zeng et al. 2023). In addition, safflower petals are extremely rich in flavonoids, in terms of both types and content; among others, hydroxy–safflower yellow A is effective in alleviating inflammation, has antitumor properties, promotes blood circulation, and can help to resolve blood stasis (Cullerne et al. 2021; Deng et al. 2023; Ngyen et al. 2021). Due to its high linoleic acid and flavonoid content, safflower is considered a valuable medicinal and edible crop in China (Yingqi et al. 2019, 2022).

The *APETALA2/ethylene‐responsive factor (AP2/ERF)* transcription factor superfamily is widely distributed in plants and plays crucial roles in plant growth, development, and stress responses (Hu, Zhang, and Guo 2022). Additionally, *AP2/ERF* transcription factors are involved in regulating the biosynthesis of secondary metabolites essential for plant defense and adaptation (Usha Kiran et al. 2015). According to sequence similarity and the number of *AP2/ERF* domains, the *AP2/ERF* superfamily can be divided into the *AP2, ERF*, and *ABI3/VP1 (RAV)*‐related families. The *ERF* family is further subdivided into the ERF and C‐repeat binding factor/dehydration‐responsive element‐binding (CBF/DREB) protein subfamilies (Lu et al. 2018).

Numerous studies have investigated the *AP2/ERF* family in various plant species, including *Arabidopsis thaliana* , rice, grapes, and *Salvia miltiorrhiza* (Bİlmez Özçinar 2022). However, limited research has been conducted on the *AP2/ERF* transcription factors in safflower. Based on the safflower genome data obtained from our previous studies (Li et al. 2020), the present study is aimed at exploring the *AP2/ERF* transcription factors in safflower and screening the transcription regulatory factors closely related to this crop. By analyzing 127 *AP2/ERF* genes, we provide foundational data for further exploration of the growth, development, and stress response mechanisms in safflower, thereby contributing to both scientific understanding and potential agricultural applications.

In this study, we identify the *AP2/ERF* transcription factor family in safflower and analyze their phylogeny, gene structure, conserved motifs, and expression patterns. The findings offer insights into the diverse functions and evolutionary relationships of *AP2/ERF* genes in safflower, laying the groundwork for future investigations into their roles in plant growth, development, and stress responses.

## 2 Materials and Methods

### 2.1 Identification and Classification of *APETALA2/Ethylene‐Responsive Factor* Genes in *C. tinctorius* L.

The sequences of *CtAP2/ERF* genes were obtained from the safflower genome database (https://safflower.scuec.edu.cn/). The hidden Markov model (HMM) file (PF00847) of the *AP2/ERF* domain was downloaded from the Pfam database (https://pfam.xfam.org/) and used as a query to search for *AP2/ERF* sequences with an *e*‐value of <1 × 10^−5^ (Usha Kiran et al. 2015). A second search was conducted using a species‐specific model with a standard *e*‐value of <1 × 10^−10^ to obtain the anticipated protein sequences (Usha Kiran et al. 2015). The sequences were then submitted to the Simple Modular Architecture Research Tool (http://smart.embl‐heidelberg.de), National Center for Biotechnology Information Conserved Domain Database (https://www.ncbi.nlm.nih.gov/Structure/bwrpsb/bwrpsb.cgi), and Pfam database to verify the presence of the *AP2* domain. Redundant sequences were removed after confirming that they belonged to the same locus.

### 2.2 Phylogenetic Analysis and Conserved Motif Identification

Multiple sequence alignment of the full‐length AP2/ERF proteins and *AP2/ERF* domains was performed using Clustal X. Phylogenetic trees were constructed using the neighbor‐joining method with 1000 bootstrap replicates in MEGA11 software. Conserved motifs in the *CtAP2/ERF* transcription factors were analyzed using the MEME motif elicitation software (Version 5.5.5) (https://meme.sdsc.edu/meme/cgi‐bin/meme.cgi) with default parameters and a maximum number of 20 motifs. The conserved structure was generated using TBtools (https://www.tbtools.com/).

### 2.3 Gene Structure and Chromosomal Localisation Analysis

The genomic DNA sequences containing 4000 bps upstream and downstream of each *AP2/ERF* gene were downloaded from the safflower genome database. The gene structure (exon–intron organization) was analyzed and visualized using TBtools. The chromosomal location of each *AP2/ERF* gene was obtained from the GigaDB website (https://gigadb.org/dataset/100613) and visualized using TBtools.

### 2.4 *Cis*‐Acting Element Analysis and Protein–Protein Interaction Prediction

The genomic DNA sequences of 2000–4000 bps upstream of the *AP2/ERF* genes were downloaded and submitted to PlantCARE (https://bio.tools/plantcare) for cis‐acting element prediction. Protein–protein interactions of the AP2/ERF proteins were predicted using the Search Tool for the Retrieval of Interacting Genes/Proteins (STRING) (https://string‐db.org/) with *A. thaliana* selected for comparative analysis and a minimum required interaction score of 0.400.

### 2.5 Expression Profile Analysis

To investigate the expression patterns of the *CtAP2/ERF* genes, high‐throughput transcriptome sequencing data from safflower roots, stems, leaves, flowers, and seeds at different developmental stages were analyzed. The expression levels of the *CtAP2/ERF* genes were estimated, normalized, and presented as fragments per kilobase of transcript per million mapped reads. Fold change (log_2_) values were calculated based on the ratio of gene expression, and heat maps were generated using TBtools.

### 2.6 Plant Material, Stress Treatments, and Quantitative Real‐Time Polymerase Chain Reaction Analysis

Safflower seedlings (variety “Yu Honghua No.1”) were grown under controlled conditions (25°C ± 2°C, 16 h light/8 h dark cycle) and subjected to various stress treatments, including 10% PEG6000, 200 mM NaCl and low temperature (4°C). Leaf samples were collected at 0, 3, 6, 12, and 24 h posttreatment, immediately frozen in liquid nitrogen, and stored at −80°C for RNA extraction.

Total RNA was isolated using the Quick RNA Isolation Kit (HuaYueYang, Beijing, China), and first‐strand cDNA was synthesized using the PrimeScriptTM RT Reagent Kit with gDNA Eraser (TaKaRa, Beijing, China) according to the manufacturer's instructions. Quantitative real‐time polymerase chain reaction (qRT‐PCR) was performed using SYBR® Green qPCR Mix (Monad, Suzhou, China) on a QIAquant 96 1.10 plex real‐time detection system (Qiagen, Germany). The qRT‐PCR conditions were as follows: 95°C for 30 s, followed by 40 cycles of 95°C for 10 s and 60°C for 30 s (see the qPCR primer list in Table 1). The Ct 60S gene was used as a reference gene for normalization. Relative expression levels were calculated using the 2^−ΔΔCT^ method (Yingqi et al. 2022). All reactions were performed in triplicate, and the results were presented as means ± standard deviation.

**TABLE 1:** qPCR sequence list.

Name	Sequence	Length
CtAH08T0201600.1‐F	AATACCGAGGGGTGGCAA	18
CtAH08T0201600.1‐R	GATCCGCGTAAAGGCTGA	18
CtAH01T0036400.1‐F	GCAGTGGTTTCTCTAGGGGA	20
CtAH01T0036400.1‐R	GTGGGTGATTTGGTCGGAT	19
CtAH02T0053100.1‐F	ACCTACGACACACCAGAAAAGG	22
CtAH02T0053100.1‐R	GGGTTGGAGGAGCAATGAA	19
CtAH08T0036500.1‐F	GGTGGTACTGGTGGGAATGT	20
CtAH08T0036500.1‐R	CTAGGGTTAGGGTTTGTGTTTG	22
CtAH04T0074600.1‐F	GCAGGAGCAACGAAAGAGAA	20
CtAH04T0074600.1‐R	CAGTAGGAAAGTTAGTAACGACGAC	25
CtAH07T0052500.1‐F	ACATCGGGATGAAAGTGTGG	20
CtAH07T0052500.1‐R	CCGGTGGATCTCTGAAAGC	19
CtAH02T0074300.1‐F	ATACTTCCCTCCTGTGCCC	19
CtAH02T0074300.1‐R	CAAACTGATTCCAACCCCTA	20
CtAH05T0095700.1‐F	CCACCAAACAAACCACCAG	19
CtAH05T0095700.1‐R	ATCCCACAAAGCCGACAC	18

## 3 Results

### 3.1 Genome‐Wide Identification and Classification of *APETALA2/Ethylene‐Responsive Factor* Genes in Safflower

A total of 127 *AP2/ERF* genes were identified in the safflower genome through HMM searches and manual curation (Table S1). Among these genes, 78 contained a single *AP2/ERF* domain and were classified into the *ERF* family, 21 belonged to the *AP2* family, 5 were assigned to the *ABI* family (containing an *AP2/ERF* domain and a B3 domain), 21 were categorized into the *AT1G* and *AT4G* families, and the remaining 17 genes were grouped into the *CRF* family. The number of *AP2/ERF* genes identified in safflower was lower than that in *A. thaliana* (147).

### 3.2 Phylogenetic Analysis and Conserved Motif Identification

**FIGURE 1:**
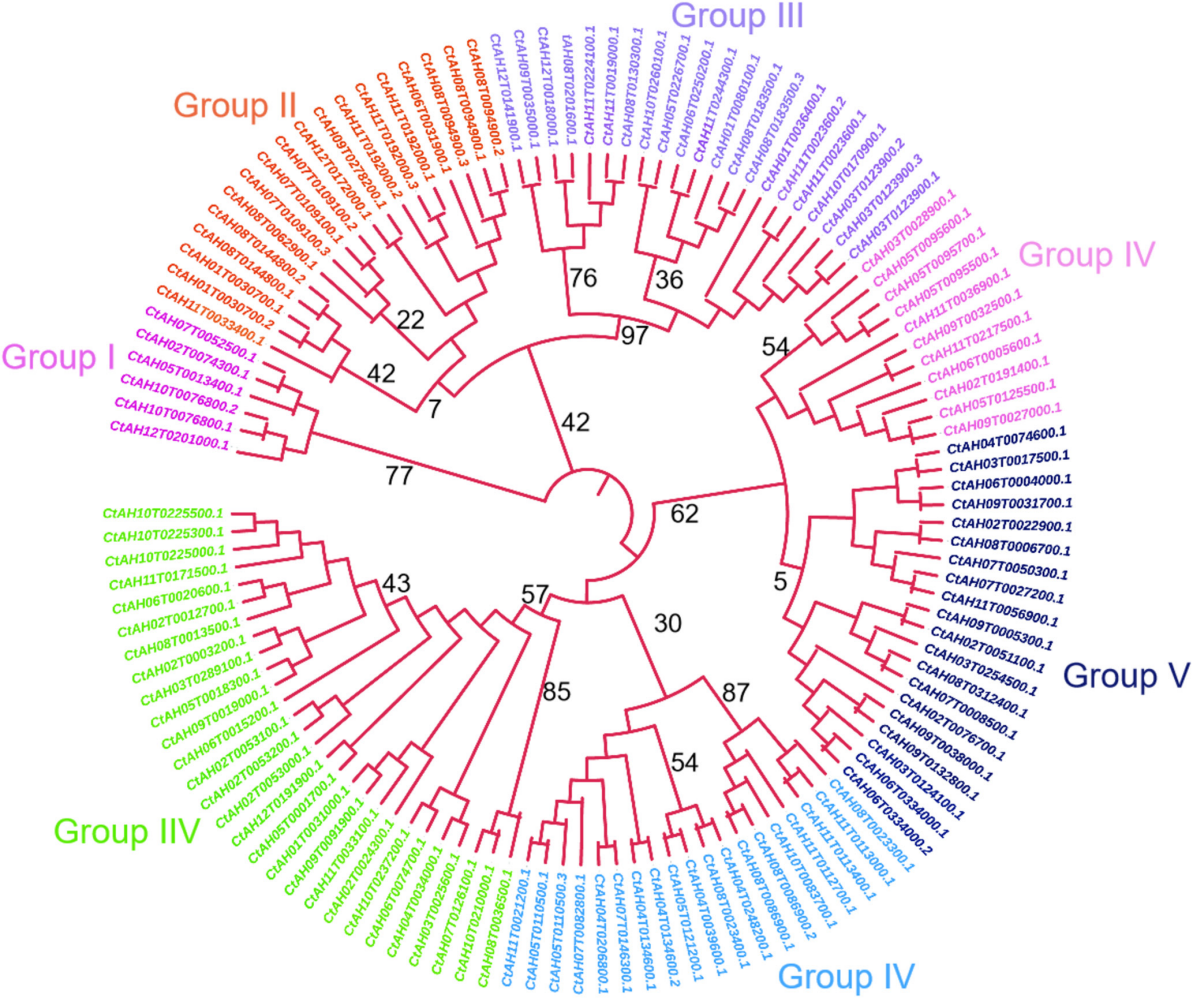
Phylogenetic analysis of the AP2/ERF proteins in safflower and *Arabidopsis thaliana* using the maximum likelihood method. Phylogenetic trees were constructed with the MEGA11 program using the AP2/ERF domain sequences. The trees were generated with Clustal X2 and MEGA11 software using the neighbor‐joining method with 1000 bootstrap replicates. According to the classification rules of the AP2/ERF genes in safflower, all AP2/ERF genes were clustered into 7 groups and 14 subgroups. DREF: DNA replication‐related element‐binding factor.

**FIGURE 2:**
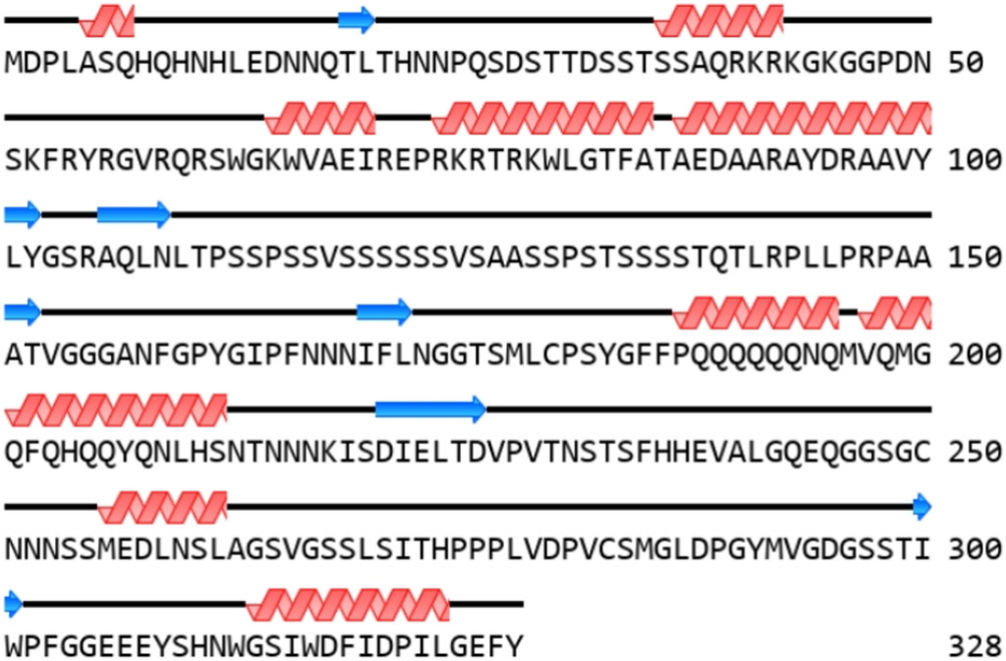
Multiple sequence alignment of conserved domains in AP2/ERF proteins of safflower.

Phylogenetic analysis of the CtAP2/ERF proteins based on the alignment of full‐length sequences and *AP2/ERF* domains revealed that the 127 genes could be classified into seven groups and 14 subgroups (Figure 1). Multiple sequence alignment of the *AP2/ERF* domain sequences showed that the basic region and two helical structures were highly conserved in most AP2/ERF proteins (Figure 2). Among the conserved amino acid residues, 12 exhibited a sequence identity > 75%, with Glu‐2, Arg‐10, and Glu‐12 being highly conserved in the basic region.

### 3.3 Gene Structure, Chromosomal Localisation, and Collinearity Analysis

**FIGURE 3:**
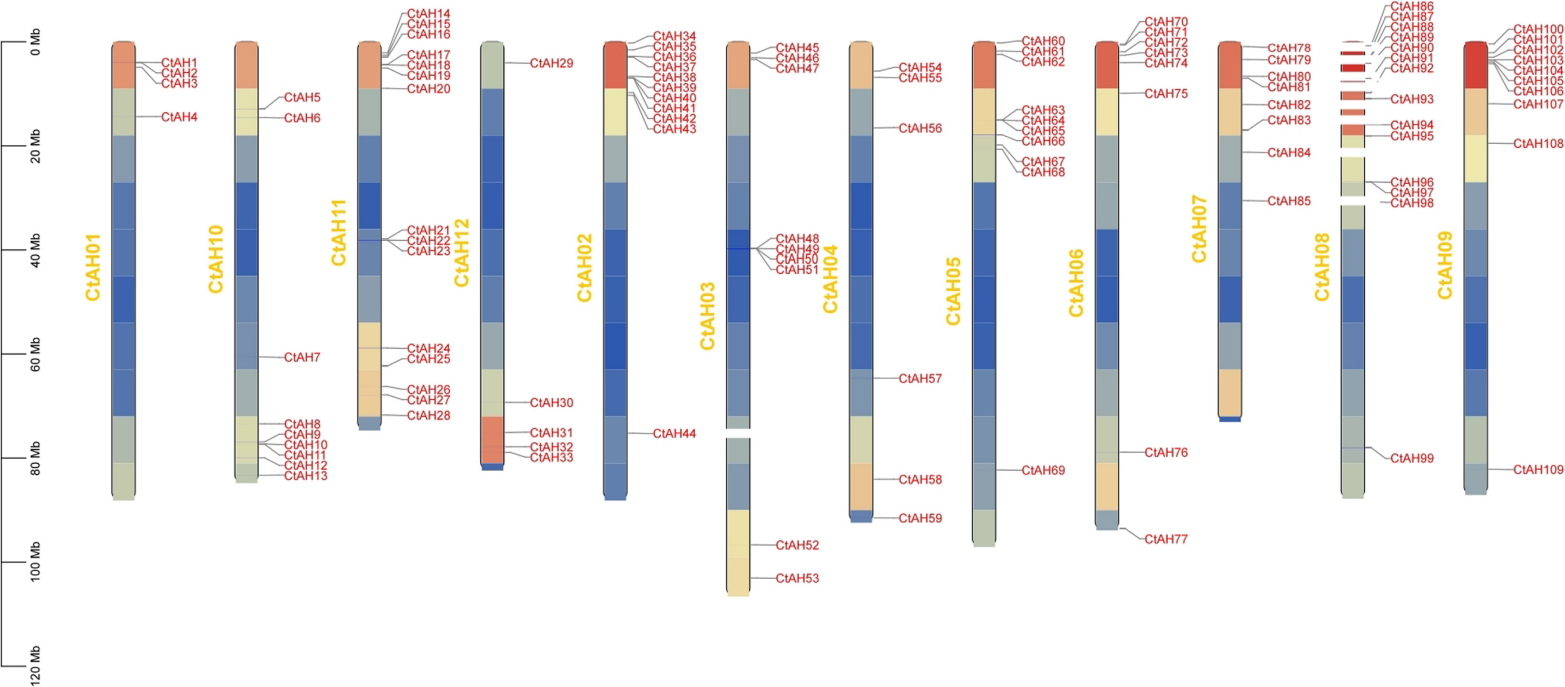
Chromosomal distribution of 127 AP2/ERF genes in safflower. The scale on the left represents the length of the safflower chromosomes in Mb.

**FIGURE 4:**
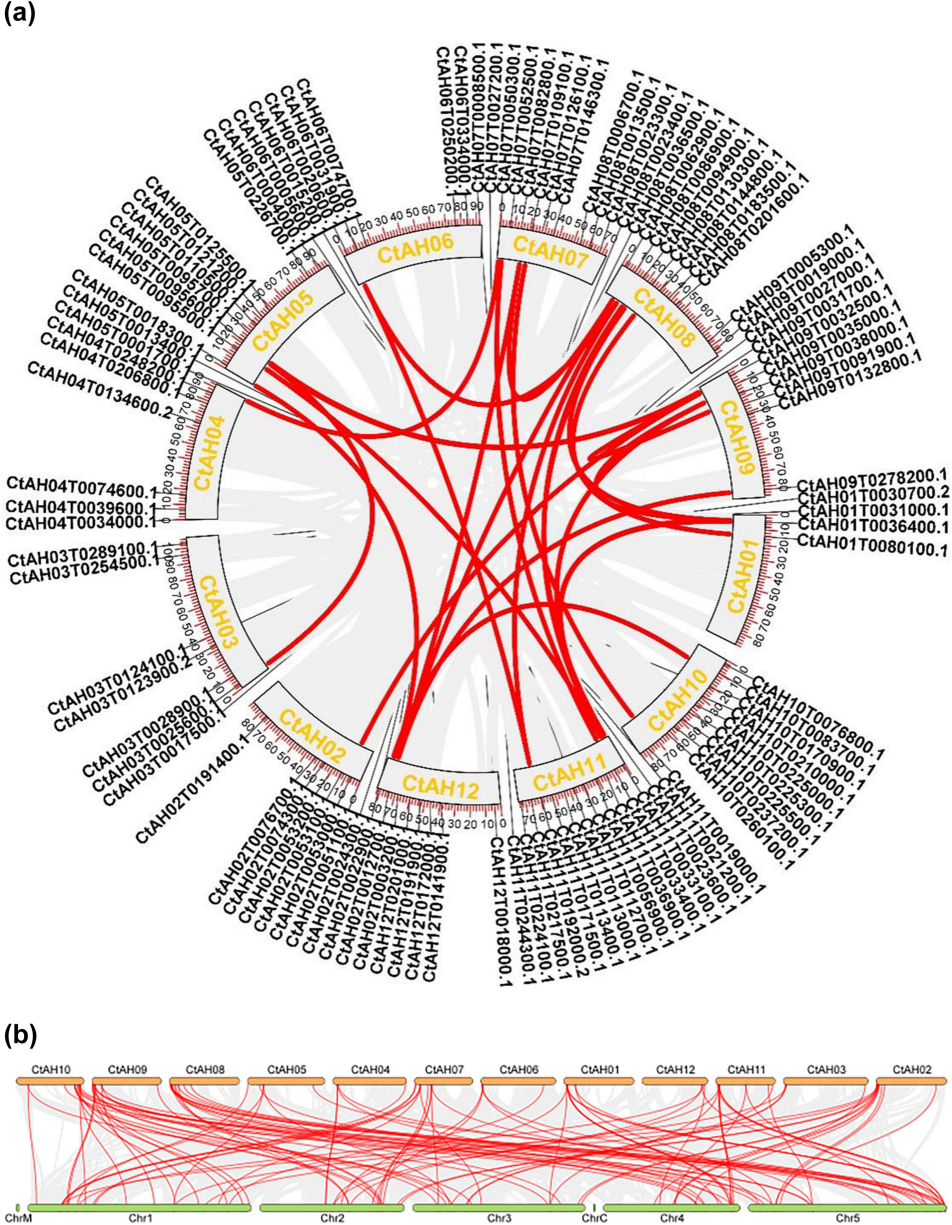
Intra and interspecies collinearity analysis of AP2/ERF genes. (a) Intraspecies collinearity analysis between safflower and *Arabidopsis thaliana* . The orange lines represent chromosomes, the red lines represent all collinear genes in safflower, and the gray lines represent collinear genes between safflower and Arabidopsis. (b) Interspecies collinearity analysis in safflower. The orange lines represent chromosomes, the red lines represent all collinear genes in safflower, and the gray lines represent collinear genes within safflower.

Analysis of the gene structure and chromosomal localisation of the *CtAP2/ERF* genes revealed that 10 genes could not be assigned to specific chromosomes, while the remaining 117 genes were unevenly distributed across the 12 safflower chromosomes (Figure 3). Chromosome 11 harbored the highest number of *CtAP2/ERF* genes (20), followed by chromosome 2 (10), while chromosome 1 contained the lowest number (5). Collinearity analysis identified 56 *AP2/ERF* genes in the safflower genome that were collinear, with 32 homologous genes in the *A. thaliana* genome (Figure 4a). Moreover, four *AP2/ERF* genes in safflower were collinear with five homologous genes in *Arabidopsis*, and three safflower *AP2/ERF* genes were collinear with three homologous genes in *Arabidopsis* (Figure 4b).

### 3.4 Expression Profile Analysis of *C. tinctorius* L. *APETALA2/Ethylene‐Responsive Factor* Genes in Different Tissues

**FIGURE 5:**
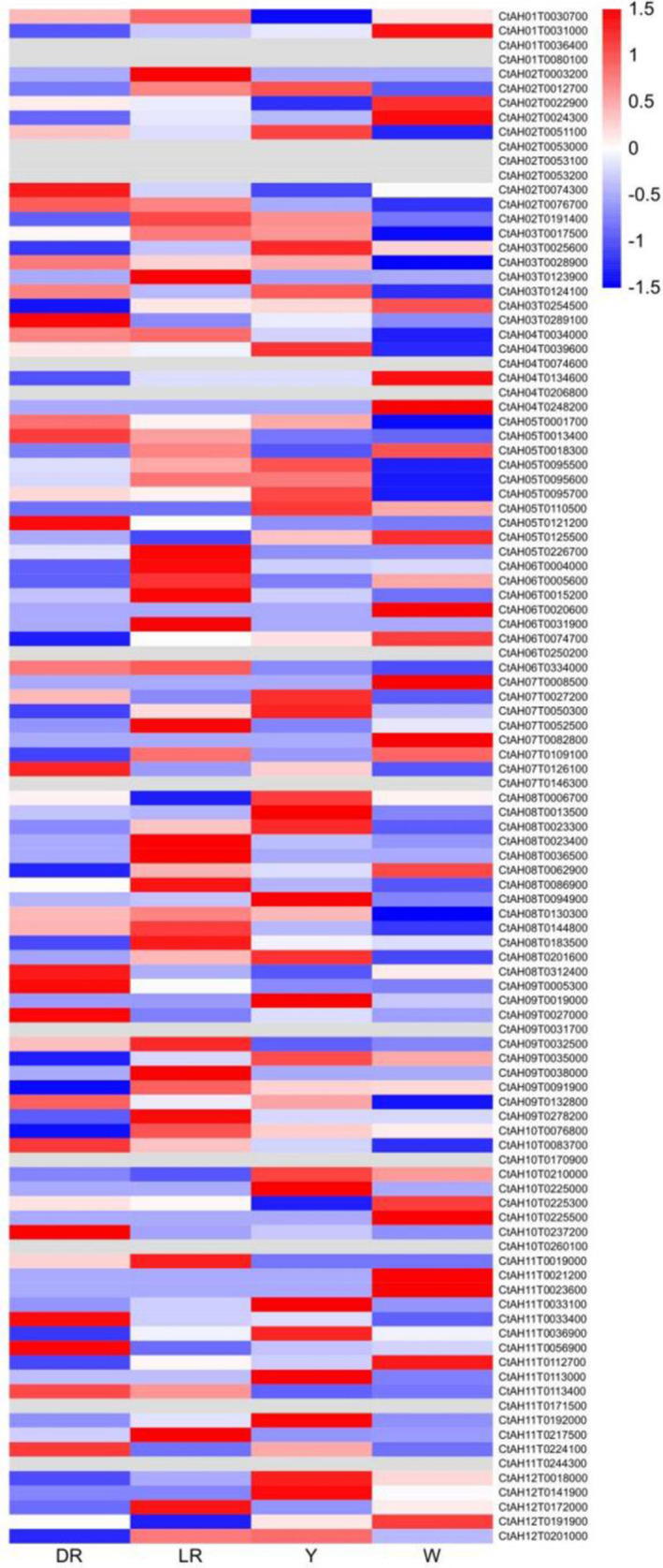
Expression profiles of the ERF family genes in different tissues of safflower. The color scale represents fragments per kilobase of transcript per million mapped reads (FPKM), with light green indicating low expression levels and red indicating high expression levels.

**FIGURE 6:**
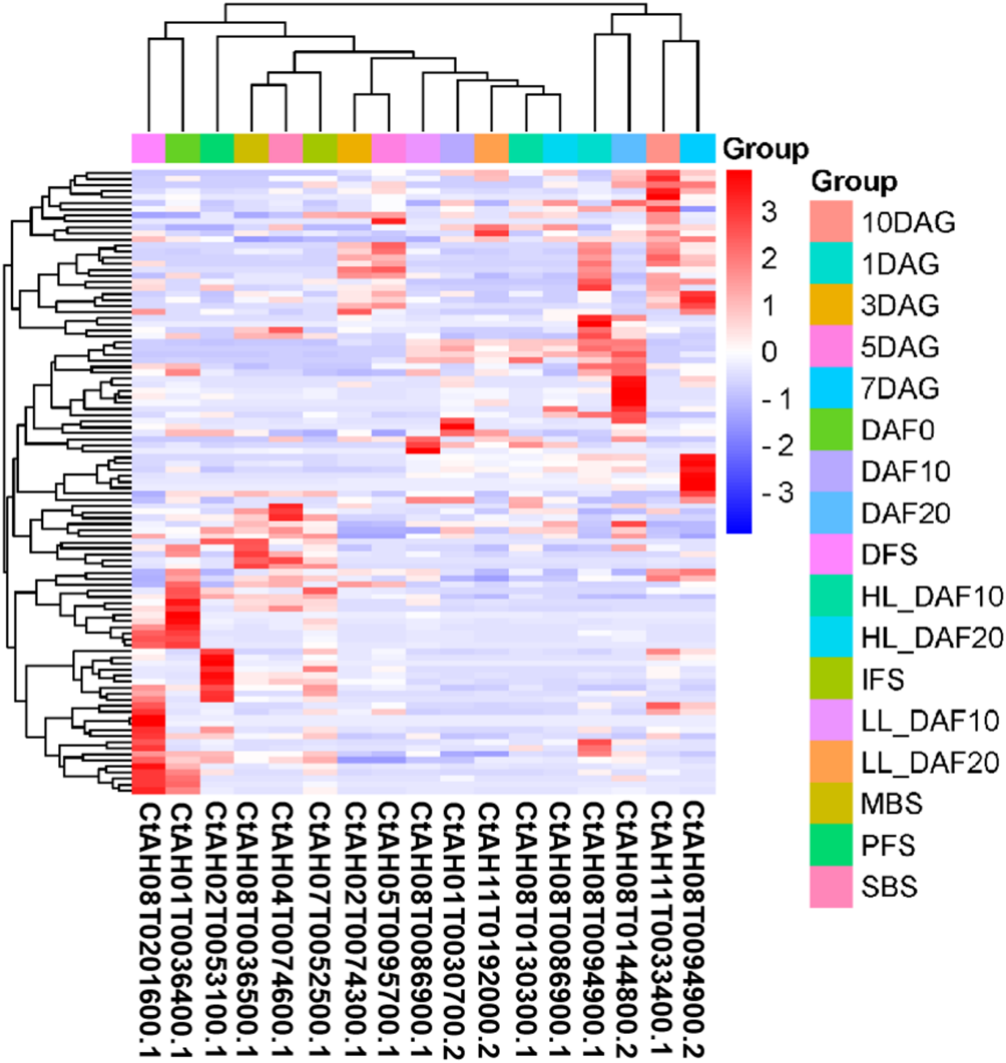
Expression patterns of the ERF family genes during five flower developmental stages in safflower (SBS, MBS, IFS, PFS, and DFS). The color scale represents log2‐transformed FPKM values, with light green indicating low expression levels and red indicating high expression levels. CtAH02T0051100 is marked in red, and the bar chart at the top right represents the normalized Z‐score value of FPKM. SBS: Small Bud Stage; MBS: Medium Bud Stage; IFS: Initial Flowering Stage; PFS: Peak Flowering Stage; DFS: Dying Flower Stage.

The expression patterns of 106 *CtAP2/ERF* genes in four different safflower tissues (roots, stems, leaves, and flowers) were analyzed using transcriptome sequencing data (Figure 5). Expression was not detected for 10 genes, while 59 genes exhibited varying degrees of specificity in different safflower varieties. Only eight genes (CtAH01T0030700, CtAH06T0334000, CtAH04T0039600, CtAH08T0023400, CtAH05T0095700, CtAH06T0005600, CtAH03T0017500, and CtAH05T0013400) were highly expressed in all four tissues (Figure 6). Furthermore, 23, 21, 15, and 9 genes were most abundantly expressed in roots, leaves, flowers, and buds, respectively (Figure 6). The expression patterns of key gene families involved in the biosynthesis processes related to growth, development, and stress tolerance in safflower were also investigated during five flower developmental stages (SBS, MBS, IFS, PFS, and DFS) (Figure 6).

### 3.5 Cis‐Acting Element Analysis and Protein–Protein Interaction Prediction

**FIGURE 7:**
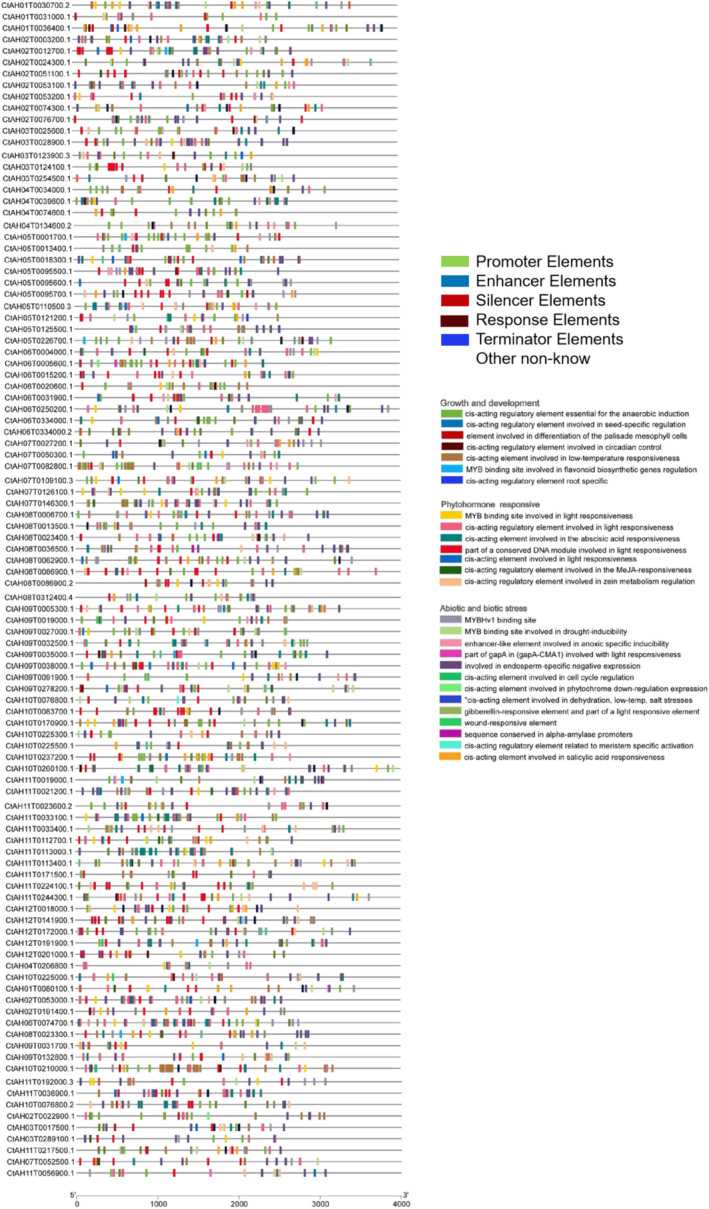
Analysis of cis‐acting elements in the promoter regions of 127 AP2/ERF genes in safflower. Potential *cis*‐acting elements in the promoter regions from −4000 bp to the start codon (ATG) of the AP2/ERF genes were predicted using TBtools. Different colors indicate elements associated with growth and development (circadian control) and stress responses (hypoxia, light, low temperature, and drought).

**FIGURE 8:**
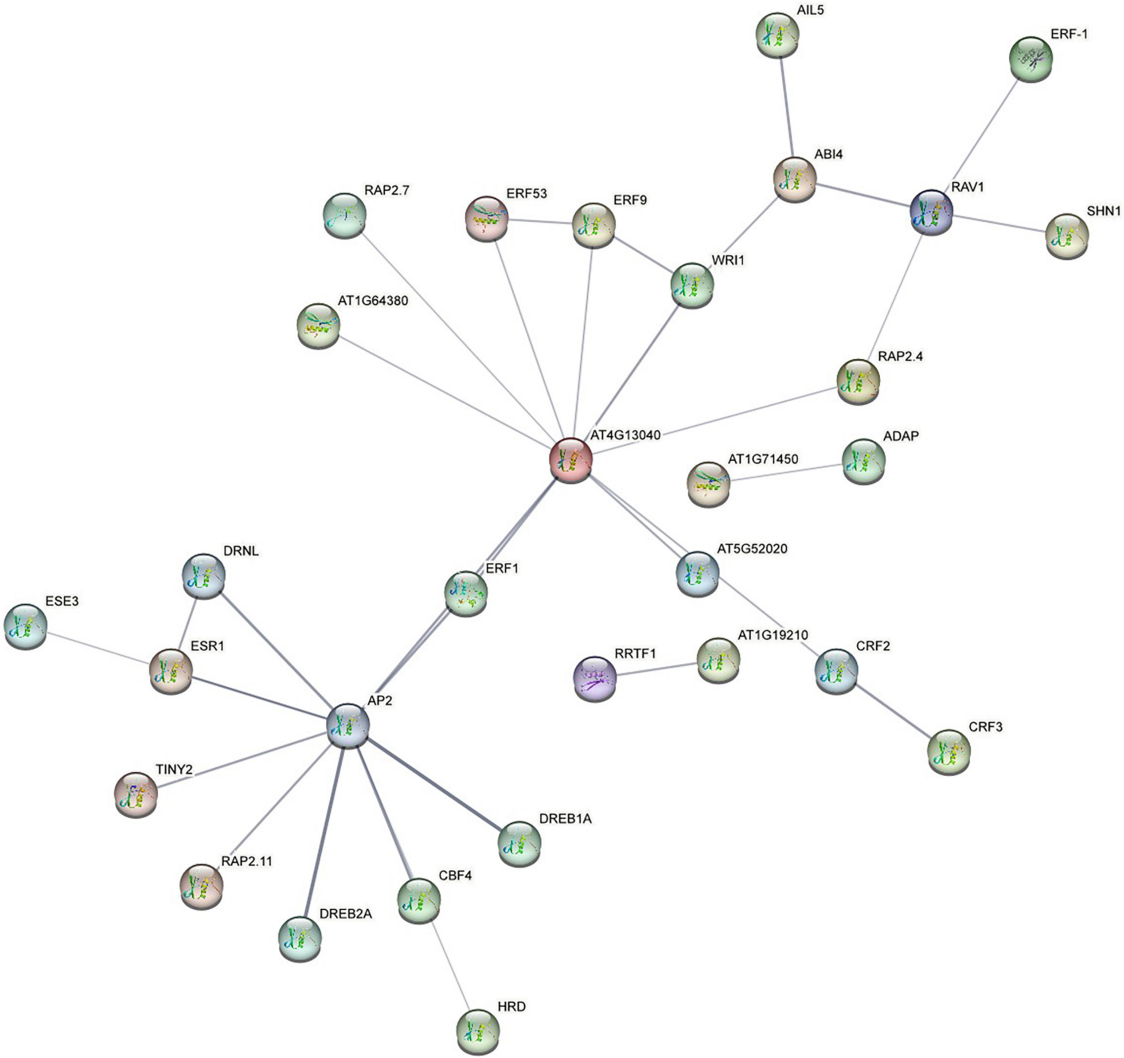
Functional regulatory network of 33 AP2/ERF proteins in safflower. Protein–protein interactions of AP2/ERF proteins were predicted using STRING (Search Tool for the Retrieval of Interacting Genes/Proteins). The cyan lines represent data from curated databases, the purple lines represent experimentally determined interactions, the green lines represent gene neighborhood, the red lines represent gene fusions, the blue lines represent gene cooccurrence, the yellow lines represent text mining, the black lines represent coexpression, and the gray lines represent protein homology.

Analysis of the *cis*‐acting elements in the promoter regions of the 127 *AP2/ERF* genes revealed that they could be divided into three main types (Figure 7). A total of 54 and 32 *AP2/ERF* genes were identified to contain *cis*‐acting elements associated with nucleic acid and transcription factor binding activities, respectively. Protein–protein interaction prediction of 33 CtAP2/ERF proteins using STRING identified seven main classes: ABI4, AP2, AT1G19210, AT4G13040, CRF2, ERF53, and RAV1 (Figure 8). Homology analysis with *Arabidopsis* protein sequences revealed that CtAH11T0033400 (homologous to TINY2) belonged to the VII(a+b) subfamily, and TINY2, a DREB protein, is involved in cold and drought tolerance. CtAH12T0191900 interacted with RRTF1, which regulates light signaling and plant hormone signaling pathways, while CtAH12T020100 (homologous to WRI1) belonged to the III(d+e) subfamily. CtAH02T0022900 was homologous to CRF3, and CtAH07T0052500 was homologous to SHN1. AT4G13040 interacted with ERF53 and ERF9, and RAV1 interacted with ABI4 and ERF‐1.

### 3.6 Expression Analysis of *C. tinctorius* L. *APETALA2/Ethylene‐Responsive Factor* Genes Under Abiotic Stress Conditions

To investigate the expression patterns of CtAP2/ERF genes in response to abiotic stress, safflower seedlings were subjected to PEG6000, NaCl, and low‐temperature treatments, and the expression levels of selected genes were analyzed using qRT‐PCR (Table 2). The results showed that after 3 h of treatment, the expression of CtAH01T0036400.1, CtAH02T0053100.1, CtAH08T0036500.1, CtAH04T0074600.1, CtAH04t0074600.1, CtAH04t0053100.1, CtAH04t0074600.1 and CtAH05T0095700.1 differed significantly compared with the control. At 6 and 12 h posttreatment, no significant differences in expression were observed for the detected genes. However, after 24 h of treatment, all genes except CtAH04T0074600.1 and CtAH02T0074300.1 showed significant changes in expression. These results indicate that safflower *AP2/ERF* genes exhibit differential expression patterns at different time points under abiotic stress conditions, with stress‐responsive genes being significantly upregulated in the short term, while their expression tends to stabilize over time.

**TABLE 2:** Identification of expression levels of related genes.

	0 h			3 h			6 h			12 h			24 h		
	RQ	SD	P	RQ	SD	P	RQ	SD	P	RQ	SD	P	RQ	SD	P
*Ct*AH08T0201600.1	1	0.04	/	0.64	0.00	0.05	1.17	0.07	0.27	0.65	0.01*	0.03*	0.22	0.03*	0.00**
*Ct*AH01T0036400.1	1	0.04		1.66	0.15	0.01*	0.08	0.00**	0.02*	0.24	0.14	0.02*	0.42	0.00	0.04*
*Ct*AH02T0053100.1	1	0.11		0.71	0.01	0.02	1.30	1.06	0.08	0.69	0.12	0.02*	0.51	0.17	0.02*
*Ct*AH08T0036500.1	1	0.12		11.15	1.09	0.01*	2.63	1.09	0.25	0.84	0.67	0.75	0.50	0.12	0.00**
*Ct*AH04T0074600.1	1	0.18		0.28	0.07	0.01*	0.24	0.04*	0.00**	0.66	0.19	0.02*	0.83	0.02*	0.37
*Ct*AH07T0052500.1	1	0.12		1.32	0.06	0.09	2.64	0.27	0.01*	1.61	0.02*	0.01*	2.81	0.43	0.01*
*Ct*AH02T0074300.1	1	0.51		0.62	0.00**	0.47	0.78	0.67	0.30	3.75	3.30	0.00**	0.91	0.10	0.79
*Ct*AH05T0095700.1	1	0.01		79.89	0.78	0.00**	79.57	8.56	0.00**	31.15	1.83	0.03*	11.60	1.98	0.01*

*Note:* Student's *t*‐test was used to determine the statistically significant levels for each treatment, where

^*^ *p* < 0.05,

^**^ *p* < 0.01.

## 4 Discussion

Safflower ( *C. tinctorius* L.), an important economic plant in the *Asteraceae* family, has petals and seeds that are widely used in food, medicine, and industry (Sardouei‐Nasab et al. 2023). The cultivation of safflowers is of great significance for agricultural development and economic growth. The *AP2/ERF* gene family, a group of transcription factors involved in plant growth, development, and stress responses, has been identified in various plants, including *A. thaliana* , cotton, peanuts, bamboo, and safflower (Tiwari 2013). Many *AP2*‐family genes have been reported as key regulatory factors in plant development, such as the *AP2* floral organ gene in flower development. In general, *DREB* transcription factors activate various dehydration/cold stress‐related genes, while *ERF* transcription factors regulate pathogen‐related genes (Liu et al. 2018; Sun et al. 2021).

In the present study, we conducted a comprehensive analysis of the *AP2/ERF* gene family in the safflower genome, providing the first detailed characterization of these transcription factors in this important crop. Through genomic analysis, we identified a total of 127 *AP2/ERF* genes, which were clustered into seven groups and 14 subgroups based on phylogenetic relationships. This classification highlights the diversity and complexity of the *AP2/ERF* gene family in safflower, suggesting potential functional divergence among the different groups and subgroups (Zhang et al. 2023; Thippeswamy et al. 2013).

To gain insights into the potential regulatory roles of *AP2/ERF* genes in safflower, we analyzed their expression patterns across different tissues. Notably, 23, 21, 15, and 9 genes exhibited the highest expression levels in roots, leaves, flowers, and buds, respectively, while only eight genes were highly expressed in all examined tissues. These distinct expression patterns indicate that *AP2/ERF* genes may have tissue‐specific functions in safflower growth and development. Furthermore, the identification of genes with consistently high expression across all tissues suggests their potential involvement in fundamental biological processes (Pfeiffer 2022; Chen et al. 2023).

*Cis*‐acting elements in the promoter regions of the *AP2/ERF* genes were analyzed to explore their potential regulatory mechanisms. Interestingly, we observed a degree of safflower specificity in the cis‐acting elements among different safflower species (Yang et al. 2022). This finding implies that the regulation of *AP2/ERF* genes may have evolved to adapt to the specific environmental conditions and physiological requirements of different safflower varieties.

Phylogenetic analysis provided valuable insights into the evolutionary relationships and potential functions of *AP2/ERF* genes in safflower. The clustering of genes into different groups and subgroups suggests that they may have diverged to acquire distinct functions during evolution. By comparing the phylogenetic relationships of *AP2/ERF* genes in safflower with those in other well‐studied plant species, such as *A. thaliana* , we can infer potential functional similarities and differences, guiding future ‘[/experimental investigations.

Protein–protein interaction prediction revealed that most AP2/ERF proteins in safflower are localized in the cell nucleus (Waadt et al. 2022), consistent with their role as transcription factors. However, a small number of AP2/ERF proteins were predicted to be distributed in chloroplasts, plasma membranes, and peroxisomes, indicating potential additional functions in these cellular compartments. Further studies are needed to validate these predicted localisations and elucidate the specific roles of AP2/ERF proteins in different cellular contexts.

The *AP2/ERF* gene family has been implicated in various aspects of plant growth, development, and stress responses. In safflower, understanding the functions of these transcription factors is crucial for developing strategies to enhance crop productivity and resilience. Our findings provide a solid foundation for future investigations into the molecular mechanisms underlying the roles of *AP2/ERF* genes in safflower. By integrating genomic, transcriptomic, and functional analyses, researchers can unravel the complex regulatory networks involving *AP2/ERF* transcription factors and their target genes, ultimately leading to the identification of key players in safflower growth, development, and stress tolerance.

In conclusion, our comprehensive analysis of the *AP2/ERF* gene family in safflower has shed light on the diversity, expression patterns, and potential functions of these important transcription factors. The results presented here provide a valuable resource for further studies aimed at elucidating the molecular mechanisms underlying safflower growth, development, and stress responses. By harnessing the knowledge gained from this study, researchers can develop targeted strategies for genetic improvement and sustainable cultivation of safflower, ultimately contributing to the advancement of this valuable crop in agriculture and industry.

## Author Contributions

(I) Conception and design: T.Z.W.

(II) Administrative support: L.D.D. and Y.Y.L.

(III) Provision of study materials or patients: L.L. and X.L.J.

(IV) Collection and assembly of data: D.W. and L.C.M.

(V) Data analysis and interpretation: T.Z.W., Y.Q., and L.H.Z.

(VI) Manuscript writing: all authors.

(VII) Final approval of manuscript: all authors.

## Ethics Statement

This study was conducted in accordance with the Declaration of Helsinki. This study was conducted with approval from the Ethics Committee of the Institute of Chinese Herbel Medicines, Henan Academy of Agricultural Sciences.

## Consent

The authors have nothing to report.

## Conflicts of Interest

The authors declare no conflicts of interest.

## Supporting information


**Data S1.** Peer review.

**Table S1.** A total of 127 CtAP2/ERF family genes.

## Data Availability

All data generated or analyzed during this study are included in this article.
