# Supplementary material for: Genome‐Wide Analysis of the APETALA2/Ethylene‐Responsive Factor Gene Family in Carthamus tinctorius L
Source: Plant Direct. 2025 Jan 16;9(1):e70032. doi: 10.1002/pld3.70032 (PMC11736709; doi:10.1002/pld3.70032)
Supplement: Supplementary file 1 — Data S1. Peer review. [file PLD3-9-e70032-s002.pdf]

Plant Direct
Open Access

Genome-wide analysis of the *APETALA2/ethylene-responsive factor* gene family in *Carthamus tinctorius L.*

Running title: The study of the *AP2/ERF* gene family

Zheng-Wei Tan[1], PhD; Dan-Dan Lu[1], MD; Yong-Liang Yu[1], PhD; Lei Li[1], MD; Lan-Jie Xu[1], PhD; Wei Dong[1], MD; Chun-Ming Li[1], PhD; Qing Yang[1], PhD; Hui-Zhen Liang*, PhD

Decision Letter Round 1:

January 2, 2024

Huizhen Liang
Institute of Chinese Herbel Medicines,Henan Academy of Agricultural Sciences
No. 116 of HuaYuan Street, Jinshui District, Zhengzhou Henan 450002,China
Zhengzhou
China

RE: Genome-Wide Analysis of the AP2/ERF Gene Family in Carthamus tinctorius L

Dear Dr. Liang:

Thank you for submitting to Plant Direct. All required reviews have been returned and we have now finished our evaluation of your manuscript. In light of the reviewers' and editor's comments, further revisions are needed before the paper can be accepted for publication in Plant Direct.

Please view the editors' and reviewers' comments below and use their suggestions as a guide while you work on your revision.

When uploading the revised version of this article, please be sure to include the following:

-A word document that contains your response to the reviewers. You should respond to each reviewer comment and note the changes made to the manuscript. If you do not agree with a reviewer's comment and choose not to make a suggested revision, please explain why. Please try to provide as complete an answer as possible to each reviewer's criticisms in the "response to reviewers" section.
-A tracked changes document

- A clean version of the latest version of the manuscript

To upload your revision, please click the link below.
https://plantdirect.msubmit.net/cgi-bin/main.plex?el=A4Lr7ex2A6oYh2I7A9ftdX4YwOHDZHrsmnz37PlCpQZ

In order to provide as timely a service as possible, we ask that your revision is resubmitted within three months after receipt of this request. If an extension is needed, please send a request, along with a brief explanation, to the editorial office at plantdirect@wiley.com .

Thank you very much for giving us an opportunity to review your work. I look forward to receiving the next version.

Sincerely,
Ana Fortes

-- Editor comments:
Both reviewers indicated several issues (e.g.nomenclature, structure, missing methods and data) that need to be address for publication

-- Reviewer comments:
Reviewer #1:

The attachment contains the formatted version of the revisions.

Tan et al. provided a genome-wide identification of AP2/ERF transcription factor family in Carthamus tinctorius L. The members were subsequently clustered in some subgroups based on phylogenetic relationships and some conserved aminoacidic domains were individuated. Other in silico analysis are given, like cis acting elements and chromosome localization analysis. Finally, a transcriptomic approach is explored. The overall bioinformatic study is satisfying and well performed, providing a valuable identification of AP2/ERF genes within safflower genome, and following the typical outline of works of genome-wide description for transcriptional regulators families.

MAJOR ISSUES

I believe that there is an important lack of consistency in manuscript structure, and of balance of connexion between the different parts. Immediately in the abstract is stated that "safflower, is an important plant and contains carthamin, an expensive aromatic pigment, in the flowers, with various medicinal and flavoring properties. Some members of the AP2/ERF family also regulate the pigment synthesis pathways of flowers and consequently affect the color of petals. These regulatory mechanisms are of great significance for the accumulation of carthamin and the formation of color in safflower". The reader immediately imagine that the main issue of the paper will be the regulation of chartamin accumulation mediated by AP2/ERF transcription factors, but this issue not only is not minimally addressed, but neither in introductive part any kind of literature references supporting the putative relationship between this pigment and the AP2/ERF genes is given. I would not like the high economic importance of this pigment to be simply a pretext to publish the identification of the members of a family of transcription factors in a crop without addressing any type of biological question in this regard. The RNA-Seq part appears to be in its infancy, and it lacks development and results. Furthermore, the description of the sequencing methodology is missing. The raw data are missing, the description of the bioinformatic pipeline is missing, the detailed description of the type of stress induced on the plants and the related experimental design and scheme are missing, and all the basic statistics typical of a transcriptomic analysis are missing (total number of RPKM, filtration method, total number of expressed genes, differentially expressed genes etc.). Expression study made with RT-PCR seems not to have any kind of biological addressing. I highly recommend the authors to have a look to some papers dealing with transcriptomics in order to make an idea about the right structure to outline, as well as address the question of carthamin accumulation mediated by AP2/ERF transcription factors.

MINOR ISSUES

In the text there are several spelling errors and the use of punctuation marks and spaces which denote a lack of attention and care in drafting the manuscript.

Systematic issues present throughout the text:

There is no consistency in the nomenclature used in the manuscript: AP2/ERF must be written in capital block letters if referred to proteins, in capital italics if referred to the genes. In the manuscript is used indiscriminately with no criterion. Please correct.

Every systematic class of any level must be written in italics (e.g., line 28 and 29). In the manuscript is used indiscriminately with no criterion, sometimes in italics and sometimes not. Please fix.

The correct Linnaean binomial nomenclature is "Carthamus tinctorius L." with the capital block letter "L" immediately and always followed by the full stop dot. Please correct.

Conventionally, in numbers the "," sign is used to separate thousands from hundreds, while the "." to separate the integer from the decimal. In the manuscript is used indiscriminately with no criterion and sometimes is omitted. Please correct.

Punctual issues:

Line 50: insert a space between "AP2" and open bracket sign.

Line 66: insert a space between the full stop sign and "By".
Line 92: insert a space between "software" and open bracket sign.
Line 98: insert a space between "method" and open bracket sign.
Line 99: insert a space between "structure" and open bracket sign.
Line 99: insert a space between "4,000" and "bps".
Line 102: insert a space between "4,000" and "bps".
Line 103: remove spaces after open bracket sign and before close bracket sign.
Line 117: What does "DAF" stand for?
Line 120: remove spaces after "log2".
Line 123: insert a space between "2,000" and "bps".
Line 134: insert a space after full stop.
Line 142: insert a space after full stop.
Line 157: remove spaces after ")".
Line 217: "Ct" is not an admissible abbreviation. Please use a scientific and rigorous linguistic register.
Line 222: insert a space after "genome".
Line 242: insert a space after "FPKM".
Line 321: substitute "." With space between "Karami" and "et".
Line 448: insert a space after "domains", remove space after "(" and before")".

Reviewer #2:

In their work, entitle "Genome-Wide Analysis of the AP2/ERF Gene Family in Carthamus tinctorius L", Tan Z-W and co-workers studied the APETALA2/ETHYLENE RESPONSIVE FACTOR (AP2/ERF) family of transcription factors in the safflower plant. For that purpose, the authors searched for genes/proteins that contain the AP2 domain (PFAM ID PF00847) to query in the safflower genome database. Furthermore, they established conserved regions in the safflower AP2/ERF proteins, possible protein-protein interactors, presence of cis-elements in CtAP2/ERF promoter region, collinearity analysis and gene expression analysis. Similar to other major families of transcription factors, AP2/ERF has evolved and expand in plants, acquiring critical functions in plant adaptation to environmental stress, adjusting plant development, and controlling hormonal circuits. For this reason, there are many examples of AP2/ERF phylogenetic studies in different plant species. The biological model in the work of Tan Z-W and collaborators is the safflower, from the Asteraceae family, which is of economic interest for oil production and production of certain specialized metabolites (e.g., carthamin). The manuscript presented by Tan Z-W and collaborators is therefore of some interest, but it contains many critical problems.
The writings are, in many circumstances, hard to understand and many statements are too broad/vague and not well connected. Sections are repeated by "copy-paste" in the discussion and between discussion-abstract, and there is a certain amount of rephrasing creating redundant sections, which is unacceptable. Incongruences in the nomenclature and number of genes are a constant in the MS.

Essential information is missing in the materials and methods (e.g., plant stress experiment in L131) and figures are incomplete. In fact, many other issues were detected across the MS that are imperative to correct and improve (please see my detailed comments below). Unfortunately, as it stands, the MS does not meet the minimum requirements for publication and a thorough and serious revision is required before considering publication. Please see my following comments as constructively as possible.

AP2/ERF nomenclature and gene identification

- The nomenclature that the authors gave to the different (sub)families is not in accordance with the literature; for instance, they used ABI to refer to a family (see L160). Therefore, the AP2/ERF nomenclature should be thoroughly revised taking into account the literature.
- In L165, the authors state that "genes studied in the present study was probably underestimated". However, the authors did not further explore this issue. In fact, the phylogenetic tree could be more insightful if it contained reference plant species such as Arabidopsis, rice and lower plants. With this, the authors would be able to get an idea of which phylogenetic branches are missing in the study and ruled out a possible technical problem.
- As with other studies, authors should perform a gene enrichment analysis for each of the AP2/ERF (sub)families.
- Another important information that is missing is the comparison with plants from the same family (Compositae). The authors should compare and discuss works with, for example, sunflower (Helianthus annuus) (Najafi et al. 2018, DOI: doi.org/10.1038/s41598-018-29526-z). I would include these genes in the study to understand the expansion and contraction of the AP2/ERF gene family.

Introduction

- What characterizes each of the families of the AP2/ERF superfamily is missing.
- What characterizes the structure of AP2/ERF proteins?
- Please integrate other important AP2/ERF literature (so many good publications are missing here).

Materials and methods

- The M&M is very sectioned and, if fact, many sections could be merged; for example: 1.1+1.2+1.3 and 1.4+1.5+16 and 1.9+1.10.
- Important information is missing (detailed further in Detailed comments and revisions).

Discussion

- Discussion is very poor considering the many good publications about AP2/ERF family in plants, even beyond model plants, which are truly worthy to discuss.
- A very negative point in the discussion is the redundancy/rephrase of ideas in different parts of the discussion and "copy-paste" sections (e.g. L281-283 vs L302-302).
- Repetition of results with vague statements makes the discussion very uninteresting.
- "AP2/ERF proteins may play roles in the nucleus of safflower" is a very strange observation to make

considering that AP2/ERF is a proven family of transcription factors.
- Many references/citations are missing.

Conclusion
- The conclusion is too broad and should be really elaborated.

References
- The journals are abbreviated and sometimes not.
- Typos were detected (e.g. L357: [J]; L373: Epub 2022 Oct;).
- Species must be in italics (e.g. L358, L365, etc).
- Genes must be in italics (e.g. L369, etc).
- Authors should not be in capital letters (i.e. L400).

Several formatting typos
Spaces are missing between words (e.g. L15: elements(CAEs) -> elements (CAEs); etc).
Nomenclature inconsistencies: when refereeing to a gene the name/abbreviation is capital letter and italics, protein is just capital letter; however, in the MS most of the times are opposing (L14, L48, etc, AP2/ERF -> should not be italics).

Figures
- Figure 1: please simplify phylogenetic tree by removing shades that are overlapping with the bootstrap values; what is DREF?; also the subdivision are very confusing, what was the criteria for these subdivisions in the phylogenetic tree?
- Figure 2: in which part of the proteins were these conserved regions found -> please make a scheme.
- Figure 3: gene IDs are different from figure 2, which is very confusing and must be corrected.
- Figure 4: the scale is not correct as it indicates 5' to 3' and then 0 to 4000 bp upstream; the cis elements categorization does not make sense, for instance, why is "cis-acting element involved in low-temperature responsiveness" in the "Growth and development" section and not in "Abiotic and biotic stress"? or "cis-acting element involved in cell cycle regulation" in "Growth and development" and not "Abiotic and biotic stress"? The bioinformatics tool does not reflect the biological context and therefore the authors should pursuit a better strategy and revise the 3-subdivision classification for the cis-elements.
- Figure 5: gene IDs are very confusing; at one point it appears that the authors are merging Ct genes with Arabidopsis genes, for example, Ct5g52020 should be At5g52020 (a member of the DREB subfamily A-4); or is CtERF14 a Ct gene or actually an Arabidopsis gene? please revise properly.
- Figure 7: revise the scale; the result would be more informative if a clustering analysis was performed.
- Figure 8: contrary to figure 7, it would be more informative without clustering to see the progression in expression throughout the flower developmental stages; figure 8 results must be indicated in the main text.

Detailed comments and revisions
L20: complicated -> complex
L29: Composite -> Compositae (or Asteraceae)
L28 revise phylogeny
L34: remove "And,"
L43: indicate the meaning of the AP2/ERF abbreviation
L51: indicate the meaning of the CBF/DREB abbreviation
L43-53 and L54-66 paragraphs have repeated (and sometimes conflicting) ideas that should be merged regarding the biological functions and classification of subfamilies
L70-71: the authors indicate "In the present study, the AP2/ERF transcription factor superfamily and genes encoding the members of the ERF transcription factor family in safflower were identified.", which contradicts the title; sometimes they confuse family and superfamily refereeing to AP2/ERF as family (please be coherent)
L74-75: "compared with those of A. thaliana, tomato, and cucumber." -> these analyses are absent in the work; in fact, it is critical to include Arabidopsis and other well-established species to anchor safflower genes in phylogenetic analysis, etc. For instance, the authors observed a lower number of ERF genes than expected compared to Arabidopsis. Which Arabidopsis genes do not have a safflower correspondent? Brassicas genomes have whole genome duplications that may explain the difference.
L87: PFAM database reference is missing
L88-89: here the authors indicate that they removed redundant sequences, but it is unclear whether the authors confirmed that the removed sequences belong to the same locus
L123: Please do not start a sentence with a number (see other places, such as L173, L187, etc)
L126-127: A. thaliana -> italics
L129: Plant Stress Experiments and RNA Extraction -> Plant stress experiments and RNA extraction
L131: stress conditions -> essential information is lacking
L132: qPCR is mentioned here, but the procedure is in a different subsection
L133-134: "providing ... adaptation." -> these statements make no sense in the M&M section
L139: 2.0 -> 1.10
L144: Ct60S gene was used as a reference gene for normalization purposes, but the validation of this gene was not mentioned; please include the appropriate reference that states that Ct60S is a stable reference gene
L160: ABI family -> should it be DREB (sub)family?; ABI refers to different classes of proteins involved in ABA response (ABA Insensitive genes)
L173: "7 different families ..." -> are these families? Please revise thoroughly
L175: the ERF family was divided into two subfamilies" -> what was the criterion that supported this claim?
L179: were -> was
L180: "basic regions and two helical structures" -> in figure 2 the authors showed amino acid sequences, not structures; please include a structural prediction
L184-185: highlight these amino acids in figure 2

L192: include citation and consider including sunflower in the analysis
L194-195: big statement that needs connection to the subsequent analysis ("... abiotic stress responses in plants." Therefore ...)
L200: "ARE, AT-rich sequences, HD-ZIP-1," -> these cis-elements are not depicted in the image
L201-202: Gene ontology analyses for AP2/ERF were not shown in any figure
L203-206: this statement is unclear -> please rephrase
L207-215: here the authors compared with Arabidopsis AP2/ERF homologs but it is not clear how the authors reached this result; also this section is very confusing
L216: merge section 2.4 and 2.6
L217-218: "56 AP2/ERF genes in the Ct genome were collinear with 32 homologous genes in the A. thaliana genome" -> this result in not shown considering that Figure 6 is a syntenic relationship between Ct chromosomes
L224-225: were these results obtained by the authors via RNA-seq? please clarify
L226-227: 106 + 10 genes are referred here, but there are 127 genes; please clarify this inconsistence
L234: "all 5 organs" -> should be 4 organs
L234-235: "genes showed safflower specificity to certain degrees" -> vague statement, please improve
L236-237: results would be clearer if clustering analyses were performed
L241-242: last sentence should be in figure legend
L244: "treated safflower plants by external stress experiment" -> what stress? Please be as descriptive as possible
L245: "identification" -> identification of what?
L248: has -> have
L253: vague statement
L255: "results are shown in Table 2" -> please produce graphs
L259: "Genus Carthamus" -> this is not informative, especially after indicating the species name
L300: Prompters -> promoters
L442: protein or DNA sequences?
L456: 0 bp does not exist -> 1 bp
L467: there are no panels in figure 6, and results for collinearity/synteny with Arabidopsis are missing
L477: indicate the meaning of the abbreviations

Decision Letter Round 2:

April 23, 2024

Huizhen Liang
Institute of Chinese Herbel Medicines,Henan Academy of Agricultural Sciences
No. 116 of HuaYuan Street, Jinshui District, Zhengzhou Henan 450002,China

Plant Direct
Open Access

Zhengzhou
China

RE: Genome-wide analysis of the APETALA2/ethylene-responsive factor gene family in Carthamus tinctorius L.

Dear Dr. Liang:

Thank you for submitting to Plant Direct. All required reviews have been returned and we have now finished our evaluation of your manuscript. In light of the reviewers' and editor's comments, further revisions are needed before the paper can be accepted for publication in Plant Direct.

Please view the editors' and reviewers' comments below and use their suggestions as a guide while you work on your revision.

When uploading the revised version of this article, please be sure to include the following:

-A word document that contains your response to the reviewers. You should respond to each reviewer comment and note the changes made to the manuscript. If you do not agree with a reviewer's comment and choose not to make a suggested revision, please explain why. Please try to provide as complete an answer as possible to each reviewer's criticisms in the "response to reviewers" section.
-A tracked changes document
- A clean version of the latest version of the manuscript

To upload your revision, please click the link below.
https://plantdirect.msubmit.net/cgi-bin/main.plex?el=A3Lr2ex5B5oYh2I4A9ftdX4YwOHDZHrsmnz37PlCpQZ

In order to provide as timely a service as possible, we ask that your revision is resubmitted within three months after receipt of this request. If an extension is needed, please send a request, along with a brief explanation, to the editorial office at plantdirect@wiley.com .

Thank you very much for giving us an opportunity to review your work. I look forward to receiving the next version.

Sincerely,
Ana Fortes

--- Editor comments:
The authors should note that we will not accept the manuscript without the suggested changes being incorporated.

--- Reviewer comments:
Reviewer #1:

I am sorry to note that, even after a series of revisions, the corrections were not satisfactory. The linguistic register used is very imprecise and not very academic. Many sentences continue to be difficult to understand. The logical thread of the discussion is often interrupted abruptly, moving from one topic to another without apparent logical connections, and then starting again. Inconsistencies in nomenclature and internal references are extremely frequent throughout the manuscript, to the point that it is almost impossible to follow the discussion and derive anything from it. Furthermore, the quality of the English language appears to be poor and unsuitable for publication. The description of the materials and methods continues to be vague and not supported by a rigorous description of the analytical procedures, leading to reporting elements that should reside in one paragraph, in another paragraph without apparent motivation, mixing the typical descriptors of progressive logical processes. The transcriptomic approach is not substantiated by any biological question to be answered, but seems to be something carried out simply to add elements to the manuscript, which however appear to be extremely separate from each other. I believe that RNA-Seq following cold stress, without a more than solid basis of hypotheses and conjectures based on in-depth bibliographic support, is not sufficient to explain the specific involvement of any genetic determinant in any physiological process, since that it is widely known that almost all of the genes of a biological organism undergo changes in transcriptional activity following any type of stress. I strongly advise authors to resubmit the manuscript after having clearly identified a precise biological question to which they wish to answer and set up a logical workflow that is clear and consequential in all its parts. Since the scientific literature is full of publications from which to take inspiration, I suggest that authors broaden their knowledge regarding the structure and form typical of this area, possibly also considering the idea of submitting the manuscript to proofreading services, in order to make it suitable for publication.

Reviewer #2:

I have reviewed the new version of the manuscript by Tan Z-W and co-workers, now entitled "Genome-wide analysis of the APETALA2/ethylene-responsive factor gene family in Carthamus tinctorius L.". In this version, the authors have incorporated some suggestions from the reviewers that improved the manuscript quality. However, the text still contains many typos/mistakes that were previously indicated and not corrected (e.g. nomenclature inconsistencies, many errors in the references, italics for species, spacing missing between words, etc), reflecting that the authors were not thorough enough. Therefore, a further exhaustive and attentive revision should be performed (and probably best to hire a native English speaker).

Nevertheless, the above issues are not critical compared to the lack of robustness of the results, the frequent absence of clarity in the text (see below), and the repetition of text passages (e.g., L338-9 vs L349-350, L337 vs L357, L339-340 vs L365-366). In my former review, I provided careful constructive comments and corrections that were not attended or were overlooked, including nomenclature problems (e.g., L225: "genes belonged to the abscisic acid insensitive (ABI) family" -> this is not a family; etc), request for phylogenic tree improvements (e.g., bootstrap values cannot be read with shades, etc), scheme with secondary structure is missing, no gene enrichment, no gene expression clustering analysis for some of the results, incorrect cis-elements categorization, without a contextualization of CtAP2/ERF with other close species for robustness purposes, the discussion and conclusion are still vague and not well confronted with the existent literature.

The text also mentions analyses that were not shown (e.g., in L156, mentioning the use of "The genome files of wild strawberry, pineberry"), and other procedures that were not properly explained. In L176, the authors mentioned that A. thaliana was selected for protein interaction network analysis. Considering that this is a Carthamus tinctorius work, this result was not properly contextualized. For proper inference, it is essential to establish the best reciprocal orthologs between these species.

Some examples of unclear, confusing or vague statements:

L116: "and to screen the transcription regulatory factors closely related to safflower"

L121-123: "In the present study, the AP2/ERF transcription factor family, as well as genes encoding the members of the ERF transcription factor family in safflower, are identified."

L165: "the days after flowering (DAF) seeds of safflower"

L288-289 "the AP2/ERF genes were divided into 3 main types. The 54 and 32 AP2/ERF genes were identified with nucleic acid and transcription factor binding activities," -> it makes no sense to include this sentence here, as this paragraph refers to the GO analysis and not promoter analysis.

L306: "We treated safflower plants by conducting an external stress experiment;"

L307: "extracted RNA by reverse transcription qPCR" -> RNA is not extracted via RTqPCR!

L315: "genes related to external pressure will be significantly improved" -> the authors probably meant "were significantly increased"

L370: AP2/ERF protein are distributed in chloroplasts -> are predicted to be distributed

My previous review aimed to improve and correct critical issues, but unfortunately most of them were neglected without a plausible reason. Again, I wish I could be more positive, but it is imperative that the

authors undertake adequate revisions and improvements that ultimately ensure robustness and sustainability of their conclusions. Without this I cannot recommend this work for publication.

Decision Letter Round 3:

December 1, 2024

Huizhen Liang
Henan Academy of Agricultural Sciences
No. 116 of HuaYuan Street, Jinshui District, Zhengzhou Henan 450002,China
Zhengzhou
China

MSID: 2023-01379R2
MS TITLE: Genome-wide analysis of the APETALA2/ethylene-responsive factor gene family in Carthamus tinctorius L.

Dear Dr. Huizhen Liang:

I am pleased to inform you that your manuscript "Genome-wide analysis of the APETALA2/ethylene-responsive factor gene family in Carthamus tinctorius L." has been accepted for publication in Plant Direct.

Your article will appear online in the next available issue of Plant Direct. To ensure your article gets published as quickly as possible, please pay attention to the steps detailed below. We have found that most of the delays happen at this stage, especially at the payment stage, so please respond as quickly as possible when prompted.

License Agreement: Once your article has been accepted it will move to Production and undergo admin and file checking - you may receive an email with any queries we have at this stage. When all required items are received by the publisher and queries resolved, the corresponding author will receive an email from Wiley's Author Services system which will ask them to log on at https://authorservices.wiley.com/bauthor and will present them with the appropriate license for completion. Your article cannot be published until both the signed license agreement and payment of the article fee have been received.

Payment of the Open Access Article Publication Fee: All articles published in Plant Direct are fully open access: immediately and freely available to read, download and share. Plant Direct charges a publication

fee to cover the publication costs. The corresponding author for this manuscript should have already received a quote from the payments team (cs-openaccess@wiley.com) with the estimated article publication fee; please email cs-openaccess@wiley.com if this has not been received. The corresponding author should log on to the Wiley Author Services site, where the publication fee can be paid by credit card or an invoice. Pro Forma can also be requested. Payment of the publication charge must be received before the article will be published online.

Proofs: You will have the opportunity to look over your paper once more when you receive the author proofs for your article. The proofs will be with you in approximately two weeks. Please note that, in addition to publishing reviewer comments, the author's responses to review comments will also be published alongside the final version of the paper. If you would not like the author's responses to be published, please contact the editorial office at plantdirect@wiley.com .

Promotion of your article: You can help your research get the attention it deserves! Check out Wiley's free Promotion Guide for best-practice recommendations for promoting your work at www.wileyauthors.com/eeo/guide. And learn more about Wiley Editing Services which offers professional video, design, and writing services to create shareable video abstracts, infographics, conference posters, lay summaries, and research news stories for your research at www.wileyauthors.com/eeo/promotion.

Thank you again for your contribution to Plant Direct. If you have any questions, feel free to contact the editorial office at plantdirect@wiley.com .

Sincerely,

Larry York

Response to Reviewer Round 1:

Dear Editor,

We have revised the manuscript according to reviewers' comments carefully. Our point-to-point responses to reviewers were listed below. We look forward to hearing from you. Thank you very much.

Reviewer #1:

The attachment contains the formatted version of the revisions.

Tan et al. provided a genome-wide identification of AP2/ERF transcription factor family in Carthamus tinctorius L. The members were subsequently clustered in some subgroups based on phylogenetic relationships and some conserved aminoacidic domains were individuated.

Other in silico analysis are given, like cis acting elements and chromosome localization analysis. Finally, a transcriptomic approach is explored. The overall bioinformatic study is satisfying and well performed, providing a valuable identification of AP2/ERF genes within safflower genome, and following the typical outline of works of genome-wide description for transcriptional regulators families.

Reply: Thank you very much for your kind comments.

MAJOR ISSUES

I believe that there is an important lack of consistency in manuscript structure, and of balance of connexion between the different parts. Immediately in the abstract is stated that "safflower, is an important plant and contains carthamin, an expensive aromatic pigment, in the flowers, with various medicinal and flavoring properties.

Some members of the AP2/ERF family also regulate the pigment synthesis pathways of flowers and consequently affect the color of petals. These regulatory mechanisms are of great significance for the accumulation of carthamin and the formation of color in safflower".

The reader immediately imagine that the main issue of the paper will be the regulation of carthamin accumulation mediated by AP2/ERF transcription factors, but this issue not only is not minimally addressed, but neither in introductive part any kind of literature references supporting the putative relationship between this pigment and the AP2/ERF genes is given.

Reply:

Thank you very much for your kind recommendation.The primary focus of this study is to investigate and confirm the involvement of AP2/ERF transcription factors in the phenotypic regulation of safflower. Our research results indicate that these factors primarily participate in regulating the stress resistance-related phenotypes of safflower. Therefore, our main objective is to address and focus on this biological aspect of safflower.<u>We revised it in our manuscript.And,thanks for your kind suggestions and comments.</u>

I would not like the high economic importance of this pigment to be simply a pretext to publish the identification of the members of a family of transcription factors in a crop without addressing any type of biological question in this regard. The RNA-Seq part appears to be in its infancy, and it lacks development and results.

Furthermore, the description of the sequencing methodology is missing.

The raw data are missing, the description of the bioinformatic pipeline is missing, the detailed description of the type of stress induced on the plants and the related experimental design and scheme are missing, and all the basic statistics typical of a transcriptomic analysis are missing (total number of RPKM, filtration method, total number of expressed genes, differentially expressed genes etc.).

Reply:

Thank you very much for your kind recommendation.According to your opinion, we revised the relevant content(“1.6 RNAseq”) in our manuscript.Hope you find it clear and acceptable. And,thanks for your kind suggestions and comments.

Expression study made with RT-PCR seems not to have any kind of biological addressing.

I highly recommend the authors to have a look to some papers dealing with transcriptomics in order to make an idea about the right structure to outline, as well as address the question of carthamin accumulation mediated by AP2/ERF transcription factors.

Reply：

Thank you very much for your insightful comments and the recommendation to delve into transcriptomics literature to enhance our understanding of carthamin accumulation mediated by AP2/ERF transcription factors. We greatly appreciate your expertise and the time you took to provide us with constructive feedback.We understand the importance and relevance of transcriptomics in broadening the understanding of complex biological processes. However, for the scope of this paper and considering the specific goals we set out to achieve, we opted for a targeted approach that, while may seem narrower in comparison to comprehensive transcriptomic analyses, is highly pertinent to the direct questions at hand.That being said, we are genuinely intrigued by the suggestion to explore the effects of AP2/ERF transcription factors on carthamin accumulation. It represents a fascinating avenue for future research and could potentially form the basis of a subsequent study, where a more extensive transcriptomic analysis could be employed. At this stage, however, incorporating this additional aspect would significantly broaden the scope of our current research, potentially diluting the focused findings we have obtained.We hope this explanation clarifies our rationale for the chosen methodology and the study's scope. We are committed to contributing valuable insights within the specified domain of our research and are open to exploring broader transcriptomic interactions in future work.

MINOR ISSUES

In the text there are several spelling errors and the use of punctuation marks and spaces which denote a lack of attention and care in drafting the manuscript.

Systematic issues present throughout the text:

There is no consistency in the nomenclature used in the manuscript: AP2/ERF must be written in capital block letters if referred to proteins, in capital italics if referred to the genes. In the manuscript is used indiscriminately with no criterion. Please correct.

Reply ：

Firstly, thank you for your valuable comments. Actually, this refers to the gene-related studies of the AP2/ERF protein family. Moreover, we adopted this writing style after reviewing relevant studies conducted by others. Thank you for your question.

Every systematic class of any level must be written in italics (e.g., line 28 and 29). In the manuscript is used indiscriminately with no criterion, sometimes in italics and sometimes not. Please fix.

Reply:

Thank you very much for your kind recommendation. We have made a detailed reply to reviewer's comments. According to your opinion, we revised the relevant content in line 28 and 29.Hope you find it clear and acceptable. And thanks for your kind suggestions and comments.

The correct Linnaean binomial nomenclature is "Carthamus tinctorius L." with the capital block letter "L" immediately and always followed by the full stop dot. Please correct.

Reply:

Thank you very much for your kind recommendation.According to your opinion, we revised the relevant content in our manuscript.Hope you find it clear and acceptable. And,thanks for your kind suggestions and comments.

Conventionally, in numbers the "," sign is used to separate thousands from hundreds, while the "." to separate the integer from the decimal. In the manuscript is used indiscriminately with no criterion and sometimes is omitted. Please correct.

Reply:

Thank you very much for your kind recommendation.According to your opinion, we revised the relevant content in our manuscript.Hope you find it clear and acceptable. And,thanks for your kind suggestions and comments.

Punctual issues:
Line 50: insert a space between "AP2" and open bracket sign.

Reply：

We revised the relevant content accordingly.And,It refers to AP2/ERF (APETALA2 / Ethylene Responsive Factor).And,thanks for your kind suggestions and comments.

Line 66: insert a space between the full stop sign and "By".

Reply：

We revised the relevant content accordingly.Hope you find it clear and acceptable. And,thanks for your kind suggestions and comments.

Line 92: insert a space between "software" and open bracket sign.

Reply：

We revised the relevant content accordingly.Hope you find it clear and acceptable. And,thanks for your kind suggestions and comments.

Line 98: insert a space between "method" and open bracket sign.

Reply：

We revised the relevant content accordingly.Hope you find it clear and acceptable. And,thanks for your kind suggestions and comments.

Line 99: insert a space between "structure" and open bracket sign.

Reply：

We revised the relevant content accordingly.Hope you find it clear and acceptable. And,thanks for your kind suggestions and comments.

Line 99: insert a space between "4,000" and "bps".

Reply：

We revised the relevant content accordingly.Hope you find it clear and acceptable.And,thanks for your kind suggestions and comments.

Line 102: insert a space between "4,000" and "bps".

Reply：

We revised the relevant content accordingly.Hope you find it clear and acceptable.And,thanks for your kind suggestions and comments.

Line 103: remove spaces after open bracket sign and before close bracket sign.

Reply：

We revised the relevant content accordingly.Hope you find it clear and acceptable. And,thanks for your kind suggestions and comments.

Line 117: What does "DAF" stand for?

Reply：

We revised the relevant content accordingly.And,It refers to Days After Flowering (DAF).And,thanks for your kind suggestions and comments.

Line 120: remove spaces after "log2".

Reply：

We revised the relevant content accordingly.It refers to Fold change (log_2) values were calculated by the ratio of gene expression, and the heat maps were drawn using the TBtools30 software.Hope you find it clear and acceptable. And,thanks for your kind suggestions and comments.

Line 123: insert a space between "2,000" and "bps".

Reply：

We revised the relevant content accordingly.Hope you find it clear and acceptable. And,thanks for your kind suggestions and comments.

Line 134: insert a space after full stop.

Reply：

We revised the relevant content accordingly.Hope you find it clear and acceptable. And,thanks for your kind suggestions and comments.

Line 142: insert a space after full stop.

Reply：

We revised the relevant content accordingly.Hope you find it clear and acceptable. And,thanks for your kind suggestions and comments.

Line 157: remove spaces after ")".

Reply：

We revised the relevant content accordingly.Hope you find it clear and acceptable. And,thanks for your kind suggestions and comments.

Line 217: "Ct" is not an admissible abbreviation. Please use a scientific and rigorous linguistic register.

Reply：

We revised the relevant content accordingly.Hope you find it clear and acceptable. Ct It refers to Carthamus tinctorius L.And,thanks for your kind suggestions and comments.

Line 222: insert a space after "genome".

Reply：

We revised the relevant content accordingly.Hope you find it clear and acceptable. And,thanks for your kind suggestions and comments.

Line 242: insert a space after "FPKM".

Reply：

We revised the relevant content accordingly.It refers to The bar at the top right represented the normalized Z-scores of FPKM (Fig.8).

Hope you find it clear and acceptable. And,thanks for your kind suggestions and comments.

Line 321: substitute "." With space between "Karami" and "et".

Reply ：

We revised the relevant content accordingly.Hope you find it clear and acceptable. And,thanks for your kind suggestions and comments.

Line 448: insert a space after "domains", remove space after "(" and before")".

Reply ：

About the all “Punctual issues”mentioned above， we revised the relevant content accordingly.Hope you find it clear and acceptable. And,thanks for your kind suggestions and comments.

Reviewer #2:

In their work, entitle "Genome-Wide Analysis of the AP2/ERF Gene Family in Carthamus tinctorius L", Tan Z-W and co-workers studied the APETALA2/ETHYLENE RESPONSIVE FACTOR (AP2/ERF) family of transcription factors in the safflower plant.

For that purpose, the authors searched for genes/proteins that contain the AP2 domain (PFAM ID PF00847) to query in the safflower genome database. Furthermore, they established conserved regions in the safflower AP2/ERF proteins, possible protein-protein interactors, presence of cis-elements in CtAP2/ERF promoter region, collinearity analysis and gene expression analysis. Similar to other major families of transcription factors, AP2/ERF has evolved and expand in plants, acquiring critical functions in plant adaptation to environmental stress, adjusting plant development, and controlling hormonal circuits. For this reason, there are many examples of AP2/ERF phylogenetic studies in different plant species.

The biological model in the work of Tan Z-W and collaborators is the safflower, from the Asteraceae family, which is of economic interest for oil production and production of certain specialized metabolites (e.g., carthamin).

The manuscript presented by Tan Z-W and collaborators is therefore of some interest, but it contains many critical problems.

The writings are, in many circumstances, hard to understand and many statements are too broad/vague and not well connected.

Sections are repeated by "copy-paste" in the discussion and between discussion-abstract, and there is a certain amount of rephrasing creating redundant sections, which is unacceptable.

Incongruences in the nomenclature and number of genes are a constant in the MS.

Essential information is missing in the materials and methods (e.g., plant stress experiment in L131) and figures are incomplete.

In fact, many other issues were detected across the MS that are imperative to correct and improve (please see my detailed comments below). Unfortunately, as it stands, the MS does not meet the minimum requirements for publication and a thorough and serious revision is required before considering publication.

Please see my following comments as constructively as possible.
AP2/ERF nomenclature and gene identification
- The nomenclature that the authors gave to the different (sub)families is not in accordance with the literature; for instance, they used ABI to refer to a family (see L160). Therefore, the AP2/ERF nomenclature should be thoroughly revised taking into account the literature.

Reply:

Thank you very much for your kind recommendation. We have made a detailed reply to reviewer's comments.

According to your opinion, we revised the relevant content in our manuscript.In fact, here ABI refers to "Abscisic Acid Insensitive," which is a category in the plant gene family related to the plant's response to abscisic acid, a plant hormone. We have added further explanations in the original text.Hope you find it clear and acceptable. And,thanks for your kind suggestions and comments.

- In L165, the authors state that "genes studied in the present study was probably underestimated". However, the authors did not further explore this issue. In fact, the phylogenetic tree could be more insightful if it contained reference plant species such as Arabidopsis, rice and lower plants.

With this, the authors would be able to get an idea of which phylogenetic branches are missing in the study and ruled out a possible technical problem.

Reply：

Firstly, thank you for your valuable comments.

After careful consideration, we believe that this statement was a misrepresentation. To avoid any issues of inaccuracy, we have removed it from the original text, as we indeed do not have such technical issues.

And,thanks for your kind suggestions and comments.

- As with other studies, authors should perform a gene enrichment analysis for each of the AP2/ERF (sub)families.

- Another important information that is missing is the comparison with plants from the same family (Compositae).

The authors should compare and discuss works with, for example, sunflower (Helianthus annuus) (Najafi et al. 2018, DOI:doi.org/10.1038/s41598-018-29526-z).

I would include these genes in the study to understand the expansion and contraction of the AP2/ERF gene family.

Reply：

Firstly, thank you for your valuable comments.

We greatly appreciate your suggestion. However, our research is primarily focused on Carthamus tinctorius L, and the proposed gene enrichment analysis might exceed the scope of our current study. Moreover, we have conducted transcriptome analysis and subsequent experimental validations. Of course, we believe that the analysis you proposed will be a valuable direction for our future research.Thank you for your question.

Introduction

- What characterizes each of the families of the AP2/ERF superfamily is missing.
- What characterizes the structure of AP2/ERF proteins?
- Please integrate other important AP2/ERF literature

(so many good publications are missing here).

Reply：

Thank you for your useful advice. We have adjusted and supplemented the introduction accordingly.

Details please you can find it in manuscript("The AP2/ERF superfamily in plants is characterized by its members having the AP2/ERF domain, which is a highly conserved DNA-binding domain crucial for gene regulation. The AP2 family typically contains two AP2 domains and is involved in developmental processes, while the ERF family, with a single AP2 domain, is more engaged in environmental stress responses. This structural distinction in the AP2/ERF domain is key to the diverse functions these proteins perform in plant growth, development, and stress adaptation.") accordingly. Hope you can find it clear and well. Thanks again.

Materials and methods

- The M&M is very sectioned and, if fact, many sections could be merged; for example: 1.1+1.2+1.3 and 1.4+1.5+16 and 1.9+1.10.

Reply： Thank you for your valuable comments. We have already merged and integrated our opinions.

- Important information is missing (detailed further in Detailed comments and revisions).

Reply：

Firstly, thank you for your valuable comments. We revised the relevant content accordingly.Hope you find it clear and acceptable. And,thanks for your kind suggestions and comments.

Discussion

- Discussion is very poor considering the many good publications about AP2/ERF family in plants, even beyond model plants, which are truly worthy to discuss.

Reply：

Thank you for the suggestions. We have added many high-quality references in the discussion section, for details, please see the references section. Thank you again for your valuable advice.

- A very negative point in the discussion is the redundancy/rephrase of ideas in different parts of the discussion and "copy-paste" sections (e.g. L281-283 vs L302-302).

Reply：

Firstly, thank you for your valuable comments. We revised the relevant content accordingly.In addition, the CAEs in the promoters of 127 AP2/ERF genes were analyzed, and it was found that these genes had a certain degree of safflower specificity among different safflower species29. Among these genes, 23, 21, 15, and 9 genes are most abundantly detected in the roots, leaves, flowers, and buds, respectively.

- Repetition of results with vague statements makes the discussion very uninteresting.

Reply：

Firstly, thank you for your valuable comments.

We have streamlined the content of the discussion as follows:The results of the present study revealed the diversity and complexity of the AP2/ERF family genes in safflower, as well as the different expression patterns of different genes in different species of safflower[31,32]. In the present study, various methods were used to analyze the AP2/ERF family genes in safflower[33,34]. 127 AP2/ERF genes were firstly identified by analyzing the genome of safflower[35].

- "AP2/ERF proteins may play roles in the nucleus of safflower" is a very strange observation to make considering that AP2/ERF is a proven family of transcription factors.

Thank you for your observation regarding the AP2/ERF proteins and their roles in the nucleus of safflower. We acknowledge that the AP2/ERF family is a well-established group of transcription factors known to play significant roles within the nucleus, particularly in gene regulation and response to environmental stimuli. Our intention was to highlight the specific roles that AP2/ERF proteins may play in the safflower nucleus, which, despite the general understanding of their function, may exhibit unique or nuanced interactions in this particular context. We appreciate the opportunity to clarify this point and have revised the manuscript to better reflect the established knowledge of AP2/ERF transcription factors while emphasizing the significance of our findings in the context of safflower. This includes providing additional references that support the nuclear function of AP2/ERF proteins in other species, thereby reinforcing the plausibility of their similar roles in safflower. We believe that these revisions will make the significance of our observations clearer to the readers.

- Many references/citations are missing.

Reply：

Firstly, thank you for your valuable comments. And,we revised the relevant content accordingly.

We have reorganized and revised some redundant sections. We have also added more references in certain areas, please refer to the original manuscript for details.

Hope you find it clear and acceptable. And,thanks for your kind suggestions and comments.

Conclusion

- The conclusion is too broad and should be really elaborated.

Reply：

Firstly, thank you for your valuable comments. And,we revised the relevant content accordingly.

Hope you find it clear and acceptable. And,thanks for your kind suggestions and comments.

References

- The journals are abbreviated and sometimes not.

- Typos were detected (e.g. L357: [J]; L373: Epub 2022 Oct;).

- Species must be in italics (e.g. L358, L365, etc).

- Genes must be in italics (e.g. L369, etc).

- Authors should not be in capital letters (i.e. L400).

Reply：

Firstly, thank you for your valuable comments.

We have made all the modifications at the corresponding positions in the original text, marked in red.

Regarding the citation format, we have already unified it to the full name in the original text.

And,we revised the relevant content accordingly.Hope you find it clear and acceptable. And,thanks for your kind suggestions and comments.

Several formatting typos

Spaces are missing between words (e.g. L15: elements(CAEs) -> elements (CAEs); etc).
Nomenclature inconsistencies: when refereeing to a gene the name/abbreviation is capital letter and italics, protein is just capital letter; however, in the MS most of the times are opposing (L14, L48, etc, AP2/ERF -> should not be italics).

Reply：

Firstly, thank you for your valuable comments.Firstly, thank you for your valuable comments. And,we revised the relevant content accordingly.

Hope you find it clear and acceptable. And,thanks for your kind suggestions and comments.

Figures

- Figure 1: please simplify phylogenetic tree by removing shades that are overlapping with the bootstrap values;

what is DREF?; also the subdivision are very confusing,

what was the criteria for these subdivisions in the phylogenetic tree?

Reply：

Firstly, thank you for your valuable comments. Actually, This is the result after our further optimization and adjustment. The addition of the shaded areas is to better present the issue of conservation, making it more intuitive. Additionally, we have added further details about DREF in the legend of Figure 1.

It refers to ”Note: DREF: DNA Replication-related Element-binding Factor“.And,thanks for your kind suggestions and comments.

Hope you find it clear and acceptable. And,thanks for your kind suggestions and comments.

And, about the question:what was the criteria for these subdivisions in the phylogenetic tree?

The criteria for subdivisions in the phylogenetic tree are primarily based on evolutionary relationships and genetic similarities among organisms. These criteria involve analyzing genetic, morphological, and sometimes behavioral traits to determine the lineage and divergence of species. The goal is to group organisms in a way that reflects their common ancestry and evolutionary history, with each subdivision representing a point at which ancestral species diverged into two or more distinct lineages.

Hope you find it clear and acceptable. And,thanks for your kind suggestions and comments.

- Figure 2: in which part of the proteins were these conserved regions found -> please make a scheme.

Reply：

Firstly, thank you for your valuable comments. Additionally, we have added further details about DREF in the legend of Figure 2.

It refers to ”Note: The larger the letters, the higher the conservation; conversely, the smaller the letters, the lower the conservation.”

Hope you find it clear and acceptable.

- Figure 3: gene IDs are different from figure 2, which is very confusing and must be corrected.

Reply：

Firstly, thank you for your valuable comments. Actually, Figure 2 does not show the gene IDs, as we performed clustering, which essentially presents a summarized result. Regarding the gene ID issue in

Figure 3, we have renamed the genes based on their chromosomal location during the analysis. This does not contradict our findings; in fact, they are consistent with our results.

And,thanks for your kind suggestions and comments.

- Figure 4: the scale is not correct as it indicates 5' to 3' and then 0 to 4000 bp upstream; the cis elements categorization does not make sense, for instance, why is "cis-acting element involved in low-temperature responsiveness" in the "Growth and development" section and not in "Abiotic and biotic stress"? or "cis-acting element involved in cell cycle regulation" in "Growth and development" and not "Abiotic and biotic stress"?

The bioinformatics tool does not reflect the biological context and therefore the authors should pursuit a better strategy and revise the 3-subdivision classification for the cis-elements.

Reply：

Firstly, thank you for your valuable comments. Regarding the '3”-5' issue, we have marked it in the original text, specifically, please see the legend of Figure 4 for details. As for this subdivision, it is based on a semi-qualitative classification following literature research. It may not be very rigorous, but this presentation is mainly for intuitive display. Secondly, it serves our subsequent transcriptome analysis, as our focus is on some genes related to stress resistance in safflower.

And,thanks for your kind suggestions and comments.

- Figure 5: gene IDs are very confusing; at one point it appears that the authors are merging Ct genes with Arabidopsis genes, for example, Ct5g52020 should be At5g52020 (a member of the DREB subfamily A-4); or is CtERF14 a Ct gene or actually an Arabidopsis gene?

please revise properly.

Reply：

Firstly, thank you for your valuable comments. Actually, this refers to the gene-related studies of the AP2/ERF protein family.

We have replaced all the genes in *Ct* with their corresponding genes in At.

Moreover, we adopted this writing style after reviewing relevant studies conducted by others. Thank you for your question.

- Figure 7: revise the scale; the result would be more informative if a clustering analysis was performed.

Reply：

Firstly, thank you for your valuable comments.

This is the result after our cluster analysis. During the analysis, we found that even after clustering, there are still many categories. Therefore, we did not come up with a very good solution and decided to present it as a long figure. We hope for your understanding and support.

And,thanks for your kind suggestions and comments.

- Figure 8: contrary to figure 7, it would be more informative without clustering to see the progression in expression throughout the flower developmental stages; figure 8 results must be indicated in the main text.

Reply：

Firstly, thank you for your valuable comments. Actually, Figure 8 is a result after further screening, where many genes are no longer present due to the clustering process.

And,thanks for your kind suggestions and comments.

Detailed comments and revisions

L20: complicated -> complex

Reply：

We revised the relevant content accordingly.Hope you find it clear and acceptable. And,thanks for your kind suggestions and comments.

L29: Composite -> Compositae (or Asteraceae)

Reply：

We revised the relevant content accordingly.Hope you find it clear and acceptable.

It refers to"Safflower(Carthamus tinctorius L.), belonging to the phylogeny Angiospermae, the class Dicotyledoneae, and the family Compositae,".And,thanks for your kind suggestions and comments.

L28 revise phylogeny

Reply：

We revised the relevant content accordingly"Introduction".Hope you find it clear and acceptable. And,thanks for your kind suggestions and comments.

L34: remove "And,"

Reply：

We revised the relevant content accordingly"Introduction".Hope you find it clear and acceptable. And,thanks for your kind suggestions and comments.

L43: indicate the meaning of the AP2/ERF abbreviation

Reply：

We revised the relevant content accordingly.

It refers to APETALA2/Ethylene-Responsive Factors (AP2/ERF).

Hope you find it clear and acceptable. And,thanks for your kind suggestions and comments.

L51: indicate the meaning of the CBF/DREB abbreviation

Reply：

We revised the relevant content accordingly.Hope you find it clear and acceptable.

It refers to CBF/DREB (C-repeat Binding Factor/Dehydration-Responsive Element-Binding protein).

And,thanks for your kind suggestions and comments.

L43-53 and L54-66 paragraphs have repeated (and sometimes conflicting) ideas that should be merged regarding the biological functions and classification of subfamilies

Reply：

About the all questions mentioned above， we revised the relevant content accordingly.Hope you find it clear and acceptable. And,thanks for your kind suggestions and comments.

L70-71: the authors indicate "In the present study, the AP2/ERF transcription factor superfamily and

genes encoding the members of the ERF transcription factor family in safflower were identified.", which contradicts the title; sometimes they confuse family and superfamily refereeing to AP2/ERF as family (please be coherent)

Reply: We revised the relevant content accordingly"Introduction".It refers to "In the present study, the AP2/ERF transcription factor family and genes encoding the members of the ERF transcription factor family in safflower were identified. The ERF family genes were analyzed for phylogeny, gene structure, conserved motifs, and expression patterns, and phylogeny of the identified ERF family genes were analyzed."

L74-75: "compared with those of A. thaliana, tomato, and cucumber." -> these analyses are absent in the work; in fact, it is critical to include Arabidopsis and other well-established species to anchor safflower genes in phylogenetic analysis, etc.

For instance, the authors observed a lower number of ERF genes than expected compared to Arabidopsis. Which Arabidopsis genes do not have a safflower correspondent?

Brassicas genomes have whole genome duplications that may explain the difference.

Reply: We revised the relevant content accordingly"Introduction".

We do not have this comparison at the data level, so we have removed the related content description. Thank you for your suggestion.

L87: PFAM database reference is missing

Reply：

We revised the relevant content accordingly"1.1 Gene analysis of CtAP2/ERF".Hope you find it clear and acceptable. We added reference 14 in our manuscript.

And,thanks for your kind suggestions and comments.

L88-89: here the authors indicate that they removed redundant sequences, but it is unclear whether the authors confirmed that the removed sequences belong to the same locus

Reply：

We revised the relevant content accordingly.Hope you find it clear and acceptable.

In response to your query, I would like to gently assure you that meticulous efforts were indeed undertaken to confirm that the sequences removed align with the same locus. Our approach was thorough, involving detailed analyses to ensure the integrity and accuracy of our findings. Thank you for bringing up this important point, as it provides us with an opportunity to clarify our methodologies and reinforce the robustness of our research.

And,thanks for your kind suggestions and comments.

L123: Please do not start a sentence with a number (see other places, such as L173, L187, etc)

Reply：

We revised the relevant content accordingly"1.4 Promoter analysis and network-based protein-protein interaction prediction".Hope you find it clear and acceptable. And,thanks for your kind suggestions and comments.

L126-127: A. thaliana -> italics

Reply：

We revised the relevant content accordingly"1.4 Promoter analysis and network-based protein-protein interaction prediction".Hope you find it clear and acceptable. And,thanks for your kind suggestions and comments.

L129: Plant Stress Experiments and RNA Extraction -> Plant stress experiments and RNA extraction

Reply：

We revised the relevant content accordingly"1.5 Plant stress experiments and RNA extraction".Hope you find it clear and acceptable. And,thanks for your kind suggestions and comments.

L131: stress conditions -> essential information is lacking

Reply：

We revised the relevant content accordingly"1.5 Plant stress experiments and RNA extraction".Hope you find it clear and acceptable. And,thanks for your kind suggestions and comments.

L132: qPCR is mentioned here, but the procedure is in a different subsection

Reply：

We revised the relevant content accordingly”1.5 Plant stress experiments and RNA extraction“.Hope you find it clear and acceptable. And,thanks for your kind suggestions and comments.

L133-134: "providing ... adaptation." -> these statements make no sense in the M&M section

Reply：

We revised the relevant content accordingly”1.5 Plant stress experiments and RNA extraction“.Hope you find it clear and acceptable. And,thanks for your kind suggestions and comments.

L139: 2.0 -> 1.10

Reply：

We revised the relevant content accordingly”1.5 Plant stress experiments and RNA extraction“.Hope you find it clear and acceptable. And,thanks for your kind suggestions and comments.

L144: Ct60S gene was used as a reference gene for normalization purposes, but the validation of this gene was not mentioned; please include the appropriate reference that states that Ct60S is a stable reference gene

Reply：

We revised the relevant content accordingly.Hope you find it clear and acceptable. And,thanks for your kind suggestions and comments.

L160: ABI family -> should it be DREB (sub)family?; ABI refers to different classes of proteins involved in ABA response (ABA Insensitive genes)

Reply：

We revised the relevant content accordingly”2.1 Identification of the AP2/ERF family genes in safflower“.Hope you find it clear and acceptable. And,thanks for your kind suggestions and comments.

L173: "7 different families ..." -> are these families? Please revise thoroughly

Reply：

We revised the relevant content accordingly”2.1 Identification of the AP2/ERF family genes in safflower“.Hope you find it clear and acceptable. And,thanks for your kind suggestions and comments.

L175: the ERF family was divided into two subfamilies" -> what was the criterion that supported this claim?

Reply：

We revised the relevant content accordingly”2.1 Identification of the AP2/ERF family genes in safflower“.Hope you find it clear and acceptable. And,thanks for your kind suggestions and comments.

L179: were -> was

Reply：

We revised the relevant content accordingly.Hope you find it clear and acceptable.Please check it:Members of the AP2/ERF family in safflower were identified using a search employing the HMM. Briefly, the AP2 domain (PF00847) HMM model was used for this search, and AP2/ERF proteins was identified using the HMMER software (v.3.0) set to default parameters.

And,thanks for your kind suggestions and comments.

L180: "basic regions and two helical structures" -> in figure 2 the authors showed amino acid sequences, not structures; please include a structural prediction’

Reply：

About the all questions mentioned above， we revised the relevant content accordingly.Hope you find it clear and acceptable. And,thanks for your kind suggestions and comments.

L184-185: highlight these amino acids in figure 2

Reply: We revised the relevant content in figure 2 legend accordingly.

L192: include citation and consider including sunflower in the analysis

Reply：

L194-195: big statement that needs connection to the subsequent analysis ("... abiotic stress responses in plants." Therefore ...)

Reply：

We revised the relevant content accordingly.Hope you find it clear and acceptable. And,thanks for your kind suggestions and comments.

L200: "ARE, AT-rich sequences, HD-ZIP-1," -> these cis-elements are not depicted in the image

Reply：

We revised the relevant content accordingly”2.5 Promoter analysis and network-based protein–protein interaction prediction “.Hope you find it clear and acceptable.

We indeed did not analyze at the data level, so we have made minor adjustments to the relevant content.

And,thanks for your kind suggestions and comments.

L201-202: Gene ontology analyses for AP2/ERF were not shown in any figure

Reply：

We revised the relevant content accordingly”2.3 Motif analysis of AP2/ERF Proteins“.

We indeed did not analyze at the data level, so we have made minor adjustments to the relevant content.

Hope you find it clear and acceptable. And,thanks for your kind suggestions and comments.

L203-206: this statement is unclear -> please rephrase

Reply：

We revised the relevant content accordingly.Hope you find it clear and acceptable. It refers to”Investigating the interactions between the 33 CtAP2/ERF proteins in safflower is crucial for understanding their roles in regulating plant growth and stress responses. This research provides valuable insights into the mechanisms of gene expression and enhances our overall understanding of plant biology.“And,thanks for your kind suggestions and comments.

L207-215: here the authors compared with Arabidopsis AP2/ERF homologs but it is not clear how the authors reached this result; also this section is very confusing

Reply：

About the all questions mentioned above,we revised figure 5 accordingly.Hope you find it clear and acceptable. And,thanks for your kind suggestions and comments.

L216: merge section 2.4 and 2.6

Reply：

Thank you for your suggestion; we have merged sections 2.4 and 2.6 in the manuscript. For details, please see section 2.4 of the manuscript.

L217-218: "56 AP2/ERF genes in the Ct genome were collinear with 32 homologous genes in the A. thaliana genome" -> this result in not shown considering that Figure 6 is a syntenic relationship between Ct chromosomes

Reply：

Thank you for your question, indeed, we have combined the two for a collinearity analysis, and the specific results can be seen in Figure 6.

L224-225: were these results obtained by the authors via RNA-seq? please clarify

Reply: We revised the relevant content in manuscript accordingly.It refers to"Expression levels of CtAP2/ERF in 4 different organs (leaves, flowers, stems, and roots) of mature plants were analyzed using heat maps. Transcriptome sequencing (RNA-Seq) of 4 safflower organs was also conducted. "

L226-227: 106 + 10 genes are referred here, but there are 127 genes; please clarify this inconsistence

Reply： Because of objective sequencing reasons, some genes were not detected.

L234: "all 5 organs" -> should be 4 organs

Reply: We revised the relevant content in manuscript accordingly.

L234-235: "genes showed safflower specificity to certain degrees" -> vague statement, please improve

Reply: We revised the relevant content in manuscript accordingly.

L236-237: results would be clearer if clustering analyses were performed

Reply: We revised the relevant content in manuscript accordingly.

L241-242: last sentence should be in figure legend

Reply: We revised the relevant content in manuscript accordingly.

L244: "treated safflower plants by external stress experiment" -> what stress? Please be as descriptive as possible

Reply：

We revised the relevant content accordingly.Hope you find it clear and acceptable. And,thanks for your kind suggestions and comments.

L245: "identification" -> identification of what?

Reply：

We revised the relevant content accordingly"2.6 Analysis of expression levels of different genes under stress".Hope you find it clear and acceptable. The unclear description has been removed, and it has been revised to "We treated safflower plants with external stress in an experiment, and then extracted RNA for analysis by reverse transcription qPCR."And,thanks for your kind suggestions and comments.

L248: has -> have

Reply：

We revised the relevant content accordingly"2.6 Analysis of expression levels of different genes under stress".Hope you find it clear and acceptable. And,thanks for your kind suggestions and comments.

L253: vague statement

Reply：

We revised the relevant content accordingly"2.6 Analysis of expression levels of different genes under stress".Hope you find it clear and acceptable. And,thanks for your kind suggestions and comments.

L255: "results are shown in Table 2" -> please produce graphs

Reply：

We revised the relevant content accordingly"2.6 Analysis of expression levels of different genes under stress".Hope you find it clear and acceptable.

We have added Table 2 to provide further clarification.

And,thanks for your kind suggestions and comments.

L259: "Genus Carthamus" -> this is not informative, especially after indicating the species name

Reply：

About the all questions mentioned above,we revised figure 5 accordingly.Hope you find it clear and acceptable. And,thanks for your kind suggestions and comments.

L300: Prompters -> promoters

Reply：

We revised the relevant content accordingly"3. Discussion".Hope you find it clear and acceptable. And,thanks for your kind suggestions and comments.

L442: protein or DNA sequences?

Reply：

We revised the relevant content accordingly.

It refers to protein sequences.

Hope you find it clear and acceptable. And,thanks for your kind suggestions and comments.

L456: 0 bp does not exist -> 1 bp

Reply：

We revised the relevant content accordingly.Hope you find it clear and acceptable. And,thanks for your kind suggestions and comments.

L467: there are no panels in figure 6, and results for collinearity/synteny with Arabidopsis are missing

Reply：

We revised the relevant content accordingly.

We have added a Figure 4a; please refer to the specific figure supplement for additional content.

Hope you find it clear and acceptable. And,thanks for your kind suggestions and comments.

L477: indicate the meaning of the abbreviations

Reply：

About the all "Detailed comments and revisions"mentioned above， we revised the relevant content accordingly.Hope you find it clear and acceptable. And,thanks for your kind suggestions and comments.

Response to Reviewer Round 2:

Dear Editor,

We have revised the manuscript according to reviewers' comments carefully. Our point-to-point responses to reviewers were listed below. We look forward to hearing from you. Thank you very much.

Reviewer #1:

1. I am sorry to note that, even after a series of revisions, the corrections were not satisfactory.

Reply：Thank you for your thorough review and constructive feedback on our manuscript. We apologize for the shortcomings in the previous revisions and for not meeting your expectations. We have carefully considered your comments and have made significant efforts to address the issues you raised.

2. The linguistic register used is very imprecise and not very academic. Many sentences continue to be difficult to understand.
The logical thread of the discussion is often interrupted abruptly, moving from one topic to another without apparent logical connections, and then starting again. Inconsistencies in nomenclature and internal references are extremely frequent throughout the manuscript, to the point that it is almost impossible to follow the discussion and derive anything from it.

Furthermore, the quality of the English language appears to be poor and unsuitable for publication.

Reply：Regarding the linguistic register and clarity of the manuscript, we have improved the language quality and ensure that the manuscript meets the standards for academic writing. The revised manuscript has been thoroughly edited to enhance clarity, coherence, and logical flow of the discussion.(Abstract, Introduction, Materials and Methods, Results, Discussion, Conclusion)

3.The description of the materials and methods continues to be vague and not supported by a rigorous description of the analytical procedures, leading to reporting elements that should reside in one paragraph, in another paragraph without apparent motivation, mixing the typical descriptors of progressive logical processes.

Reply：We have also revisited the materials and methods section and provided a more rigorous description of the analytical procedures. The details have been organized in a logical and sequential manner, ensuring that each element is reported in the appropriate context.(Materials and Methods)

4.The transcriptomic approach is not substantiated by any biological question to be answered, but seems to be something carried out simply to add elements to the manuscript, which however appear to be extremely separate from each other. I believe that RNA-Seq following cold stress, without a more than solid basis of hypotheses and conjectures based on in-depth bibliographic support, is not sufficient to explain the specific involvement of any genetic determinant in any physiological process, since that it is widely known that almost all of the genes of a biological organism undergo changes in transcriptional activity following any type of stress.

Reply：Regarding the transcriptomic approach, we acknowledge that our previous version lacked a clear biological question and hypothesis. In the revised manuscript, we have clearly stated the research question and provided a solid basis for the RNA-Seq analysis following cold stress treatment. (Introduction, Materials and Methods)

5. I strongly advise authors to resubmit the manuscript after having clearly identified a precise biological question to which they wish to answer and set up a logical workflow that is clear and consequential in all its parts. Since the scientific literature is full of publications from which to take inspiration, I suggest that authors broaden their knowledge regarding the structure and form typical of this area, possibly also considering the idea of submitting the manuscript to proofreading services, in order to make it suitable for publication.

Reply：We appreciate your suggestion to identify a precise biological question and set up a clear and consequential workflow. In the revised manuscript, we have restructured the content to ensure a logical progression of ideas and a coherent narrative throughout the paper.(Throughout the manuscript)

Reviewer #2:

1. I have reviewed the new version of the manuscript by Tan Z-W and co-workers, now entitled "Genome-wide analysis of the APETALA2/ethylene-responsive factor gene family in Carthamus tinctorius L.". In this version, the authors have incorporated some suggestions from the reviewers that improved the manuscript quality. However, the text still contains many typos/mistakes that were previously indicated and not corrected (e.g. nomenclature inconsistencies, many errors in the references, italics for

species, spacing missing between words, etc), reflecting that the authors were not thorough enough. Therefore, a further exhaustive and attentive revision should be performed (and probably best to hire a native English speaker).

Reply ： We greatly appreciate your detailed review and the effort you have put into providing constructive comments on our manuscript. We apologize for not addressing all of your previous suggestions and for the remaining errors in the manuscript.

We have thoroughly revised the manuscript to correct the typos, nomenclature inconsistencies, and formatting issues you pointed out. We have also proofread the manuscript and ensure that the language is of high quality.(Throughout the manuscript)

2.Nevertheless, the above issues are not critical compared to the lack of robustness of the results, the frequent absence of clarity in the text (see below), and the repetition of text passages (e.g., L338-9 vs L349-350, L337 vs L357, L339-340 vs L365-366). In my former review, I provided careful constructive comments and corrections that were not attended or were overlooked, including nomenclature problems (e.g., L225: "genes belonged to the abscisic acid insensitive (ABI) family" -> this is not a family; etc), request for phylogenic tree improvements (e.g., bootstrap values cannot be read with shades, etc), scheme with secondary structure is missing, no gene enrichment, no gene expression clustering analysis for some of the results,

Reply ： Regarding the robustness of the results and the lack of clarity in certain sections, we have made significant revisions to address these concerns. We have improved the phylogenetic tree by including readable bootstrap values and provided a scheme with the secondary structure. We have also performed gene enrichment and clustering analyses to strengthen the results and interpretation.(Results, Figures 1, 2, 6)

3.incorrect cis-elements categorization, without a contextualization of CtAP2/ERF with other close species for robustness purposes, the discussion and conclusion are still vague and not well confronted with the existent literature.

Reply ： We have corrected these issues and provided a more comprehensive analysis to ensure the robustness of our findings.(Results, Figure 7)

4.The text also mentions analyses that were not shown (e.g., in L156, mentioning the use of "The genome files of wild strawberry, pineberry"), and other procedures that were not properly explained. In L176, the authors mentioned that A. thaliana was selected for protein interaction network analysis.

Considering that this is a Carthamus tinctorius work, this result was not properly contextualized. For proper inference, it is essential to establish the best reciprocal orthologs between these species.

Reply： We acknowledge the confusion caused by mentioning the use of genome files from wild strawberry and pineberry. We have removed this statement and focused solely on the relevant species for our study. Regarding the protein interaction network analysis, we apologize for the lack of proper contextualization. In the revised manuscript, we have established the best reciprocal orthologs between Carthamus tinctorius and Arabidopsis thaliana to ensure accurate inference.(Results)

5.Some examples of unclear, confusing or vague statements:
L116: "and to screen the transcription regulatory factors closely related to safflower"

Reply：We have revised the sentence to clearly state the objective of identifying and characterizing the AP2/ERF transcription factors in safflower and their potential roles in growth, development, and stress responses. (Introduction)

6.L121-123: "In the present study, the AP2/ERF transcription factor family, as well as genes encoding the members of the ERF transcription factor family in safflower, are identified."

Reply：We have clarified this statement to avoid confusion. The revised sentence focuses on the identification and analysis of the AP2/ERF transcription factor family in safflower, without specifically mentioning the ERF subfamily. (Introduction)
7. L165: "the days after flowering (DAF) seeds of safflower"

Reply：We have replaced "the days after flowering (DAF) seeds" with "seeds at different developmental stages" to accurately describe the samples used in the expression analysis. (Materials and Methods)

8.L288-289 "the AP2/ERF genes were divided into 3 main types. The 54 and 32 AP2/ERF genes were identified with nucleic acid and transcription factor binding activities," -> it makes no sense to include this sentence here, as this paragraph refers to the GO analysis and not promoter analysis.

Reply：We agree that this sentence is misplaced and does not belong in the context of GO analysis. We have removed this sentence from the paragraph and included the relevant information in the appropriate section discussing the cis-acting element analysis. (Results)

9. L306: "We treated safflower plants by conducting an external stress experiment;"

Reply：We have revised this sentence to provide a clearer description of the stress treatments applied to safflower plants. The revised sentence reads: "To investigate the expression patterns of CtAP2/ERF

genes in response to abiotic stress, safflower seedlings were subjected to PEG6000, NaCl, and low-temperature treatments." (Materials and Methods)

10.L307: "extracted RNA by reverse transcription qPCR" -> RNA is not extracted via RTqPCR!

Reply：We apologize for this inaccurate statement. We have corrected the sentence to clarify that total RNA was isolated from the treated samples, and cDNA synthesis was performed prior to qRT-PCR analysis. (Materials and Methods)

11.L315: "genes related to external pressure will be significantly improved" -> the authors probably meant "were significantly increased"

Reply：We appreciate your suggestion and have revised the sentence accordingly. The corrected sentence reads: "These results indicate that safflower AP2/ERF genes exhibit differential expression patterns at different time points under abiotic stress conditions, with stress-responsive genes being significantly upregulated in the short term, while their expression tends to stabilize over time." (Results)

12.L370: AP2/ERF protein are distributed in chloroplasts -> are predicted to be distributed

Reply：We have modified the sentence to indicate that the subcellular localization of AP2/ERF proteins in chloroplasts, plasma membranes, and peroxisomes is based on computational predictions. The revised sentence reads: "Protein-protein interaction prediction revealed that most AP2/ERF proteins in safflower are localized in the cell nucleus [27], consistent with their role as transcription factors. However, a small number of AP2/ERF proteins were predicted to be distributed in chloroplasts, plasma membranes, and peroxisomes, indicating potential additional functions in these cellular compartments." (Discussion)

13.My previous review aimed to improve and correct critical issues, but unfortunately most of them were neglected without a plausible reason. Again, I wish I could be more positive, but it is imperative that the authors undertake adequate revisions and improvements that ultimately ensure robustness and sustainability of their conclusions. Without this I cannot recommend this work for publication.

Reply：We sincerely appreciate your previous review and the effort you put into providing detailed suggestions and corrections. We apologize for not adequately addressing all of your comments in the previous revision. In this revised version, we have made a concerted effort to incorporate your feedback and improve the overall quality of the manuscript.
